# Supplementary material for: An Efficient Bayesian Method for Estimating the Degree of the Skewness of X Chromosome Inactivation Based on the Mixture of General Pedigrees and Unrelated Females
Source: Biomolecules. 2023 Mar 16;13(3):543. doi: 10.3390/biom13030543 (PMC10046098; doi:10.3390/biom13030543)
Supplement: Supplementary file 1 [file biomolecules-13-00543-s001.zip › biomolecules-2230608-supplementary.pdf]

Supplementary Materials for

An Efficient Bayesian Method for Estimating the Degree of the Skewness of X Chromosome Inactivation Based on the Mixture of General Pedigrees and Unrelated Females

Yi-Fan Kong [1,2,†], Shi-Zhu Li [1,2,†], Kai-Wen Wang [1,2], Bin Zhu [1,2], Yu-Xin Yuan [1,2], Meng-Kai Li [1,2] and Ji-Yuan Zhou [1,2,*]

[1] Department of Biostatistics, State Key Laboratory of Organ Failure Research, Ministry of Education, and Guangdong Provincial Key Laboratory of Tropical Disease Research, School of Public Health, Southern Medical University, Guangzhou 510515, China; kyf990213@i.smu.edu.cn (Y.-F.K.); joey91@i.smu.edu.cn (S.-Z.L.); wen199807@i.smu.edu.cn (K.-W.W.); z20172233020@smu.edu.cn (B.Z.); 3177042030@i.smu.edu.cn (Y.-X.Y); 3167042035@i.smu.edu.cn (M.-K.L.)

[2] Guangdong-Hong Kong-Macao Joint Laboratory for Contaminants Exposure and Health, Guangzhou 510006, China

* Correspondence: zhoujy@smu.edu.cn

† These authors contributed equally to this work.

An Efficient Bayesian Method for Estimating the Degree of the Skewness of X Chromosome Inactivation Based on the Mixture of General Pedigrees and Unrelated Females

Kong *et al.*

Supplementary Appendices SA-SC

Supplementary Tables S1-S15

Supplementary Figures S1-S28

Supplementary References

Supplementary Appendices

Supplementary Appendix SA. Using the EVD to Speed up the Posterior Sampling Process for Quantitative Traits

According to Runcie and Crawford [1], we use the EVD to speed up the posterior sampling process for quantitative traits. Firstly, we decompose the genetic relatedness matrix $\boldsymbol{\varphi}$ into $\boldsymbol{Q}^T\boldsymbol{\Sigma}\boldsymbol{Q}$ by EVD, where $\boldsymbol{Q}$ is a matrix formed by the eigenvectors of the matrix $\boldsymbol{\varphi}$, and $\boldsymbol{\Sigma} = diag(\lambda_1, \lambda_2, \dots, \lambda_i, \dots, \lambda_{n_f})$ is the diagonal matrix formed by the eigenvalues λ_i's of the matrix $\boldsymbol{\varphi}$. Next, premultiply both sides of expression (5) in the main text by $\boldsymbol{Q}$ to rotate the model, and the expression becomes

$$\boldsymbol{Y}^* \sim MVN(\beta\gamma\boldsymbol{X}_1^* + \beta(2-\gamma)\boldsymbol{X}_2^* + \boldsymbol{Z}^*\boldsymbol{a}, \sigma_g^2\boldsymbol{Q}\boldsymbol{\varphi}\boldsymbol{Q}^T + \sigma_e^2\boldsymbol{I}_{n_f\times n_f}),$$

where $\boldsymbol{Y}^* = \boldsymbol{QY}$, $\boldsymbol{X}_1^* = \boldsymbol{Q}\boldsymbol{X}_1 = (X_{11}^*, X_{12}^*, \dots, X_{1i}^*, \dots, X_{1n_f}^*)$, $\boldsymbol{X}_2^* = \boldsymbol{Q}\boldsymbol{X}_2 = (X_{21}^*, X_{22}^*, \dots, X_{2i}^*, \dots, X_{2n_f}^*)$ and $\boldsymbol{Z}^* = \boldsymbol{QZ}$. Due to $\boldsymbol{\varphi} = \boldsymbol{Q}^T\boldsymbol{\Sigma}\boldsymbol{Q}$, the variance-covariance matrix $\sigma_g^2\boldsymbol{Q}\boldsymbol{\varphi}\boldsymbol{Q}^T + \sigma_e^2\boldsymbol{I}_{n_f\times n_f}$ equals the diagonal matrix $\sigma_g^2\boldsymbol{\Sigma} + \sigma_e^2\boldsymbol{I}_{n_f\times n_f}$. Finally, we transform $\boldsymbol{Y}^*$ into a normally distributed random vector, i.e.,

$$Y_i^* \sim N(\beta\gamma X_{1i}^* + \beta(2-\gamma)X_{2i}^* + \boldsymbol{a}^T\boldsymbol{Z}_i^*, \sigma_g^2\lambda_i + \sigma_e^2). \quad \text{(S1)}$$

After the transformation, the unknown parameters are still $\boldsymbol{\theta}_1 = (\beta, \gamma, \boldsymbol{a}^T, \sigma_g, \sigma_e)^T$, while the likelihood function turns to be $\boldsymbol{L}_1^*$ based on expression (S1). The corresponding posterior distribution of $\boldsymbol{\theta}_1$ becomes $f^*(\boldsymbol{\theta}_1|\boldsymbol{X}_1^*, \boldsymbol{X}_2^*, \boldsymbol{Z}^*, \boldsymbol{\Sigma}) = \frac{f(\boldsymbol{\theta}_1)\boldsymbol{L}_1^*}{\int f(\boldsymbol{\theta}_1)\boldsymbol{L}_1^* d\boldsymbol{\theta}_1}$. Compared with $\boldsymbol{L}_1$, $\boldsymbol{L}_1^*$ is the product of multiple univariate normal distributions rather than a multivariate normal distribution, which can greatly speed up the posterior sampling process.

Supplementary Appendix SB. Using Cholesky Decomposition to Speed up the Posterior Sampling Process for Qualitative Traits

As for qualitative traits, because the posterior sampling process based on the EVD in Supplementary Appendix SA cannot be applied to a Bernoulli distribution [2], we use Cholesky decomposition to accelerate the posterior sampling process. Firstly, we decompose $\boldsymbol{\varphi}$ into $\boldsymbol{C}\boldsymbol{C}^T$ by Cholesky decomposition, where $\boldsymbol{C}$ is a lower triangular matrix. Let $\boldsymbol{h} \sim MVN(\boldsymbol{0}, \boldsymbol{I}_{n_f\times n_f})$. Note that $\sigma_g\boldsymbol{Ch}$ follows $MVN(\boldsymbol{0}, \sigma_g^2\boldsymbol{\varphi})$ and then we replace b_i in equation (7) in the main text by $(\sigma_g\boldsymbol{Ch})_i$, which is the ith element of $\sigma_g\boldsymbol{Ch}$. Finally, we rewrite η_i as follows

$$\eta_i = \beta\gamma X_{1i} + \beta(2-\gamma)X_{2i} + \boldsymbol{a}^T\boldsymbol{Z}_i + (\sigma_g\boldsymbol{Ch})_i. \quad \text{(S2)}$$

In this case, the unknown parameters are still $\boldsymbol{\theta}_2 = (\beta, \gamma, \boldsymbol{a}^T, \sigma_g)^T$, but the likelihood function becomes $\boldsymbol{L}_2^*$ based on expression (6) in the main text and equation (S2). The transformed posterior distribution of $\boldsymbol{\theta}_2$ is $f^*(\boldsymbol{\theta}_2|\boldsymbol{X}_1, \boldsymbol{X}_2, \boldsymbol{Z}, \boldsymbol{C}, \boldsymbol{h}) = \frac{f(\boldsymbol{\theta}_2)\boldsymbol{L}_2^*}{\int f(\boldsymbol{\theta}_2)\boldsymbol{L}_2^* d\boldsymbol{\theta}_2}$. Compared with $f(\boldsymbol{\theta}_2|\boldsymbol{X}_1, \boldsymbol{X}_2, \boldsymbol{Z}, \boldsymbol{\varphi})$, $f^*(\boldsymbol{\theta}_2|\boldsymbol{X}_1, \boldsymbol{X}_2, \boldsymbol{Z}, \boldsymbol{C}, \boldsymbol{h})$ contains a lower triangular matrix $\boldsymbol{C}$ instead of the high-dimensional matrix $\boldsymbol{\varphi}$, which is easier to calculate.

Supplementary Appendix SC. Simulation Study of the Type I Error Rate for Our Proposed Improved Linear Mixed Model

To simulate the type I error rate of our proposed improved linear mixed model (based on the GEMMA method) for the mixture of general pedigrees and unrelated females, we conduct the following simulation study. For general pedigrees, we generate the genotypes of n_{pf} females from the pedigrees at an X chromosomal SNP by Mendelian inheritance. The process is the same as that in the "2.7. Simulation Settings" subsection in the main text. Then, the corresponding quantitative traits of these n_{pf} females can be yielded by the following distribution

$$\boldsymbol{Y}_p \sim MVN(\beta_0 + \delta \boldsymbol{K}_p, 2\sigma_g^2 \boldsymbol{\psi}_f + \sigma_e^2 \boldsymbol{I}_{n_{pf} \times n_{pf}}),$$

i.e., we just assume that $\beta = 0$ in equation (8) in the main text for simulating the type I error rate. Similarly, for n_{If} unrelated females, we generate their genotypes $\{dd, Dd, DD\}$ by probabilities $\{(1-p_f)^2,\ 2p_f(1-p_f), p_f^2\}$ at the same X chromosomal SNP. The process is consistent with that in the "2.7. Simulation Settings" subsection in the main text. The corresponding quantitative traits of these n_{If} unrelated females can be obtained by the distribution as follows

$$\boldsymbol{Y}_I \sim MVN(\beta_0 + \delta \boldsymbol{K}_I, (\sigma_g^2 + \sigma_e^2) \boldsymbol{I}_{n_{If} \times n_{If}}).$$

Note that the variance of the polygenic effects (i.e., σ_g^2) is unrelated to our generated SNP, so $\boldsymbol{Y}_p$ and $\boldsymbol{Y}_I$ do not include the information of the SNP we want to test. We set $(N_p, n_{If}) = (150, 650)$ and $(600, 2600)$, $p_f = p_m = \{0.3, 0.1\}$, $\sigma_g^2 = \{1/3, 1\}$, $(\sigma_{e0}^2, \sigma_{e1}^2, \sigma_{e2}^2) = (1, 1, 1)$ and other parameters being consistent with the "2.7. Simulation Settings" subsection in the main text. Repeat the above process 1000 times and set the significance level of the association test to be 5%. Supplementary Table S15 displays the simulated type I error rates of the improved linear mixed model. The simulation results show that the improved linear mixed model controls the type I error rate well in all the simulated situations.

Supplementary Tables

Table S1. W_{mean}'s of the BNM, BUM, BNP, BUP, BN and BU methods under $p_f = p_m$ and $(\sigma_{e0}^2, \sigma_{e1}^2, \sigma_{e2}^2) = (1, 1, 1)$ among 500 replicates for mixed data, only general pedigrees and only unrelated females, respectively.

Trait	(N_p, n_{If})	p_f	σ_g^2	MR	Mixed Data		Pedigrees		Unrelated Females	
					BNM	BUM	BNP	BUP	BN	BU
Quantitative	(150, 650)	0.3	1/3	0	0.9751	0.9813	1.2201	1.2367	1.2017	1.2149
		0.3	1/3	0.4	1.1625	1.1727	1.3898	1.4133	1.3761	1.3974
		0.3	1	0	1.0614	1.0689	1.3336	1.3570	1.3076	1.3281
		0.3	1	0.4	1.2494	1.2615	1.4847	1.5154	1.4799	1.5084
		0.1	1/3	0	1.3950	1.4138	1.5357	1.5659	1.5304	1.5628
		0.1	1/3	0.4	1.5004	1.5320	1.6064	1.6438	1.5958	1.6301
		0.1	1	0	1.5014	1.5308	1.6002	1.6392	1.5940	1.6314
		0.1	1	0.4	1.5957	1.6336	1.6634	1.7040	1.6578	1.6979
	(600, 2600)	0.3	1/3	0	0.5355	0.5359	0.7217	0.7242	0.7206	0.7234
		0.3	1/3	0.4	0.6649	0.6678	0.8832	0.8856	0.8729	0.8765
		0.3	1	0	0.6209	0.6250	0.8600	0.8676	0.8449	0.8531
		0.3	1	0.4	0.7758	0.7836	1.0461	1.0538	1.0277	1.0371
		0.1	1/3	0	1.0084	1.0166	1.2252	1.2418	1.2249	1.2389
		0.1	1/3	0.4	1.1690	1.1854	1.3583	1.3799	1.3497	1.3690
		0.1	1	0	1.1193	1.1312	1.3362	1.3541	1.3265	1.3456
		0.1	1	0.4	1.2850	1.3036	1.4442	1.4686	1.4407	1.4647
Qualitative	(150, 650)	0.3	1/3	0	1.0762	1.0823	1.3669	1.3868	1.3623	1.3829
		0.3	1/3	0.4	1.2678	1.2822	1.5143	1.5450	1.5050	1.5327
		0.3	1	0	1.2300	1.2441	1.4987	1.5294	1.4963	1.5274
		0.3	1	0.4	1.3921	1.4146	1.5949	1.6321	1.5929	1.6301
		0.1	1/3	0	1.4563	1.4765	1.5813	1.6137	1.5622	1.5807
		0.1	1/3	0.4	1.5256	1.5461	1.6138	1.6402	1.5649	1.5821
		0.1	1	0	1.5493	1.5795	1.6447	1.6846	1.6148	1.6434
		0.1	1	0.4	1.5973	1.6273	1.6479	1.6812	1.6020	1.6134
	(600, 2600)	0.3	1/3	0	0.6764	0.6796	0.8638	0.8689	0.8361	0.8399
		0.3	1/3	0.4	0.8182	0.8229	1.0565	1.0646	1.0103	1.0167
		0.3	1	0	0.7846	0.7876	1.0307	1.0374	0.9568	0.9631
		0.3	1	0.4	0.9577	0.9660	1.2033	1.2144	1.1490	1.1598
		0.1	1/3	0	1.1459	1.1544	1.3091	1.3315	1.2820	1.2949
		0.1	1/3	0.4	1.2917	1.3086	1.4284	1.4544	1.3870	1.3938
		0.1	1	0	1.2697	1.2855	1.4057	1.4260	1.3813	1.3998
		0.1	1	0.4	1.3923	1.4163	1.4971	1.5236	1.4850	1.4986

Table S2. W_{sd}'s of the BNM, BUM, BNP, BUP, BN and BU methods under $p_f = p_m$ and $(\sigma_{e0}^2, \sigma_{e1}^2, \sigma_{e2}^2) = (1, 1, 1)$ among 500 replicates for mixed data, only general pedigrees and only unrelated females, respectively.

Trait	(N_p, n_{If})	p_f	σ_g^2	MR	Mixed Data		Pedigrees		Unrelated Females	
					BNM	BUM	BNP	BUP	BN	BU
Quantitative	(150, 650)	0.3	1/3	0	0.2568	0.2811	0.3067	0.3426	0.3066	0.3392
		0.3	1/3	0.4	0.2946	0.3250	0.3100	0.3403	0.3065	0.3383
		0.3	1	0	0.2926	0.3231	0.3180	0.3523	0.3025	0.3340
		0.3	1	0.4	0.3099	0.3432	0.2815	0.3107	0.2806	0.3101
		0.1	1/3	0	0.2797	0.3112	0.2366	0.2614	0.2279	0.2549
		0.1	1/3	0.4	0.2542	0.2825	0.2179	0.2442	0.2036	0.2261
		0.1	1	0	0.2309	0.2603	0.2136	0.2353	0.2055	0.2247
		0.1	1	0.4	0.1903	0.2110	0.1758	0.1935	0.1601	0.1794
	(600, 2600)	0.3	1/3	0	0.1229	0.1284	0.1827	0.1985	0.1794	0.1955
		0.3	1/3	0.4	0.1669	0.1793	0.2385	0.2643	0.2291	0.2528
		0.3	1	0	0.1558	0.1665	0.2412	0.2665	0.2294	0.2498
		0.3	1	0.4	0.2126	0.2317	0.2866	0.3172	0.2856	0.3124
		0.1	1/3	0	0.2979	0.3230	0.2909	0.3178	0.2858	0.3160
		0.1	1/3	0.4	0.3013	0.3298	0.2775	0.3045	0.2674	0.2976
		0.1	1	0	0.2980	0.3280	0.2918	0.3206	0.2756	0.3032
		0.1	1	0.4	0.2845	0.3104	0.2576	0.2866	0.2527	0.2794
Qualitative	(150, 650)	0.3	1/3	0	0.3023	0.3348	0.3154	0.3488	0.3021	0.3328
		0.3	1/3	0.4	0.3124	0.3443	0.2859	0.3153	0.2838	0.3133
		0.3	1	0	0.3087	0.3427	0.2738	0.3011	0.2735	0.3003
		0.3	1	0.4	0.2873	0.3204	0.2419	0.2634	0.2333	0.2555
		0.1	1/3	0	0.2792	0.3099	0.2644	0.3069	0.2434	0.2740
		0.1	1/3	0.4	0.2673	0.3094	0.2979	0.3383	0.2506	0.2845
		0.1	1	0	0.2539	0.2831	0.2490	0.2908	0.1972	0.2136
		0.1	1	0.4	0.2316	0.2625	0.2980	0.3448	0.1940	0.2209
	(600, 2600)	0.3	1/3	0	0.1642	0.1774	0.2211	0.2438	0.2179	0.2353
		0.3	1/3	0.4	0.2174	0.2393	0.2867	0.3189	0.2844	0.3083
		0.3	1	0	0.2077	0.2263	0.2672	0.2950	0.2598	0.2837
		0.3	1	0.4	0.2665	0.2935	0.2995	0.3319	0.2908	0.3226
		0.1	1/3	0	0.2965	0.3257	0.2950	0.3275	0.2790	0.3098
		0.1	1/3	0.4	0.2949	0.3224	0.2917	0.3306	0.2550	0.2811
		0.1	1	0	0.2748	0.3037	0.2863	0.3188	0.2672	0.2983
		0.1	1	0.4	0.2770	0.3057	0.2617	0.3003	0.2606	0.2814

Table S3. Mean squared errors (MSEs) of point estimates $\hat{\gamma}_{BNM}$, $\hat{\gamma}_{BUM}$, $\hat{\gamma}_{BNP}$ and $\hat{\gamma}_{BUP}$ under $(p_f, p_m) = (0.3, 0.1)$ and $(0.1, 0.3)$, and $(\sigma_{e0}^2, \sigma_{e1}^2, \sigma_{e2}^2) = (1, 1, 1)$ among 500 replicates for mixed data and only general pedigrees, respectively.

**Trait**	(N_p, n_{If})	p_f	p_m	σ_g^2	MR	**Mixed Data**		**Pedigrees**	
						$\hat{\gamma}_{BNM}$	$\hat{\gamma}_{BUM}$	$\hat{\gamma}_{BNP}$	$\hat{\gamma}_{BUP}$
Quantitative	(150, 650)	0.3	0.1	1/3	0	0.0749	0.0779	0.1278	0.1371
		0.3	0.1	1/3	0.4	0.1044	0.1093	0.1781	0.1969
		0.3	0.1	1	0	0.0912	0.0999	0.1540	0.1688
		0.3	0.1	1	0.4	0.1367	0.1487	0.2139	0.2512
		0.1	0.3	1/3	0	0.1511	0.1624	0.1989	0.2130
		0.1	0.3	1/3	0.4	0.2138	0.2226	0.2798	0.3135
		0.1	0.3	1	0	0.1841	0.2025	0.2258	0.2583
		0.1	0.3	1	0.4	0.2417	0.2614	0.3255	0.3781
	(600, 2600)	0.3	0.1	1/3	0	0.0253	0.0255	0.0422	0.0460
		0.3	0.1	1/3	0.4	0.0391	0.0409	0.0693	0.0795
		0.3	0.1	1	0	0.0335	0.0355	0.0645	0.0694
		0.3	0.1	1	0.4	0.0537	0.0557	0.0900	0.0970
		0.1	0.3	1/3	0	0.0501	0.0533	0.0754	0.0798
		0.1	0.3	1/3	0.4	0.0710	0.0752	0.1260	0.1303
		0.1	0.3	1	0	0.0647	0.0673	0.1043	0.1116
		0.1	0.3	1	0.4	0.0962	0.1025	0.1430	0.1527
Qualitative	(150, 650)	0.3	0.1	1/3	0	0.1127	0.1212	0.1871	0.2040
		0.3	0.1	1/3	0.4	0.1512	0.1687	0.2367	0.2682
		0.3	0.1	1	0	0.1268	0.1374	0.2186	0.2437
		0.3	0.1	1	0.4	0.1760	0.1958	0.2905	0.3480
		0.1	0.3	1/3	0	0.1585	0.1806	0.2530	0.3000
		0.1	0.3	1/3	0.4	0.2208	0.2432	0.3645	0.4293
		0.1	0.3	1	0	0.1866	0.2088	0.2970	0.3533
		0.1	0.3	1	0.4	0.2878	0.3090	0.4072	0.4702
	(600, 2600)	0.3	0.1	1/3	0	0.0447	0.0459	0.0672	0.0671
		0.3	0.1	1/3	0.4	0.0636	0.0673	0.0967	0.1038
		0.3	0.1	1	0	0.0500	0.0529	0.0768	0.0850
		0.3	0.1	1	0.4	0.0768	0.0856	0.1137	0.1263
		0.1	0.3	1/3	0	0.0777	0.0848	0.0947	0.1033
		0.1	0.3	1/3	0.4	0.1533	0.1643	0.1657	0.1667
		0.1	0.3	1	0	0.1100	0.1162	0.1341	0.1406
		0.1	0.3	1	0.4	0.1577	0.1744	0.1839	0.1949

Table S4. Coverage probabilities (CPs, in %) of the BNM, BUM, BNP and BUP methods under $(p_f, p_m) = (0.3, 0.1)$ and $(0.1, 0.3)$, and $(\sigma_{e0}^2, \sigma_{e1}^2, \sigma_{e2}^2) = (1, 1, 1)$ among 500 replicates for mixed data and only general pedigrees, respectively [a].

Trait	(N_p, n_{If})	p_f	p_m	σ_g^2	MR	Mixed Data		Pedigrees	
						BNM	BUM	BNP	BUP
Quantitative	(150, 650)	0.3	0.1	1/3	0	94.6	95.0	95.0	95.6
		0.3	0.1	1/3	0.4	96.0	95.6	95.0	95.8
		0.3	0.1	1	0	96.0	96.0	95.0	96.2
		0.3	0.1	1	0.4	95.8	96.4	94.4	95.2
		0.1	0.3	1/3	0	95.6	96.2	94.2	96.0
		0.1	0.3	1/3	0.4	94.2	95.6	93.8	95.2
		0.1	0.3	1	0	94.0	95.6	95.8	94.8
		0.1	0.3	1	0.4	93.2	95.6	93.8	94.6
	(600, 2600)	0.3	0.1	1/3	0	95.0	95.0	96.0	95.4
		0.3	0.1	1/3	0.4	95.2	96.0	95.2	94.8
		0.3	0.1	1	0	95.0	95.8	94.6	95.0
		0.3	0.1	1	0.4	95.2	96.0	95.8	96.2
		0.1	0.3	1/3	0	93.6	93.6	96.8	97.2
		0.1	0.3	1/3	0.4	93.8	94.4	95.2	95.4
		0.1	0.3	1	0	93.4	94.8	94.4	95.0
		0.1	0.3	1	0.4	94.8	95.4	94.8	95.6
Qualitative	(150, 650)	0.3	0.1	1/3	0	95.2	94.6	95.2	94.6
		0.3	0.1	1/3	0.4	95.6	96.4	95.0	94.2
		0.3	0.1	1	0	95.4	96.6	94.6	95.0
		0.3	0.1	1	0.4	95.0	95.4	94.4	95.8
		0.1	0.3	1/3	0	94.2	94.4	95.0	95.2
		0.1	0.3	1/3	0.4	93.6	94.8	94.8	93.4
		0.1	0.3	1	0	94.2	95.6	95.4	94.8
		0.1	0.3	1	0.4	93.8	93.8	94.0	95.4
	(600, 2600)	0.3	0.1	1/3	0	93.6	94.4	94.0	94.2
		0.3	0.1	1/3	0.4	94.4	95.0	94.8	95.0
		0.3	0.1	1	0	96.0	96.6	95.4	96.2
		0.3	0.1	1	0.4	94.4	95.0	96.0	95.6
		0.1	0.3	1/3	0	93.4	94.0	94.4	94.6
		0.1	0.3	1/3	0.4	95.8	95.2	95.8	93.4
		0.1	0.3	1	0	93.6	94.2	94.0	95.2
		0.1	0.3	1	0.4	95.4	94.0	94.0	95.6

[a] The empirical CP should be between 93.05% and 96.95% ($0.95 \pm 2 \times \sqrt{\frac{0.95\times0.05}{500}}$) with 95% probability.

Table S5. W_{median}'s of the BNM, BUM, BNP and BUP methods under $(p_f, p_m) = (0.3, 0.1)$ and $(0.1, 0.3)$, and $(\sigma_{e0}^2, \sigma_{e1}^2, \sigma_{e2}^2) = (1, 1, 1)$ among 500 replicates for mixed data and only general pedigrees, respectively.

Trait	(N_p, n_{If})	p_f	p_m	σ_g^2	MR	Mixed Data		Pedigrees	
						BNM	BUM	BNP	BUP
Quantitative	(150, 650)	0.3	0.1	1/3	0	0.9824	0.9938	1.2672	1.2750
		0.3	0.1	1/3	0.4	1.1548	1.1671	1.4265	1.4724
		0.3	0.1	1	0	1.1165	1.1474	1.3871	1.4250
		0.3	0.1	1	0.4	1.3242	1.3384	1.5774	1.6148
		0.1	0.3	1/3	0	1.3164	1.3453	1.4631	1.5022
		0.1	0.3	1/3	0.4	1.4482	1.4745	1.5970	1.6501
		0.1	0.3	1	0	1.4184	1.4531	1.5883	1.6386
		0.1	0.3	1	0.4	1.5514	1.6054	1.6752	1.7303
	(600, 2600)	0.3	0.1	1/3	0	0.5873	0.5853	0.7787	0.7784
		0.3	0.1	1/3	0.4	0.7279	0.7335	0.9416	0.9375
		0.3	0.1	1	0	0.6637	0.6666	0.9021	0.9014
		0.3	0.1	1	0.4	0.8053	0.7944	1.0715	1.0703
		0.1	0.3	1/3	0	0.7905	0.7929	1.0272	1.0371
		0.1	0.3	1/3	0.4	0.9402	0.9493	1.1837	1.2074
		0.1	0.3	1	0	0.9265	0.9359	1.1525	1.1728
		0.1	0.3	1	0.4	1.0962	1.1089	1.3127	1.3261
Qualitative	(150, 650)	0.3	0.1	1/3	0	1.1426	1.1499	1.4582	1.4816
		0.3	0.1	1/3	0.4	1.3411	1.3849	1.6259	1.6762
		0.3	0.1	1	0	1.3290	1.3494	1.6153	1.6647
		0.3	0.1	1	0.4	1.4702	1.5183	1.6864	1.7389
		0.1	0.3	1/3	0	1.3350	1.3558	1.6041	1.6548
		0.1	0.3	1/3	0.4	1.5017	1.5530	1.6713	1.7315
		0.1	0.3	1	0	1.4739	1.5126	1.6839	1.7327
		0.1	0.3	1	0.4	1.5691	1.6143	1.7181	1.7685
	(600, 2600)	0.3	0.1	1/3	0	0.7311	0.7328	0.9000	0.9150
		0.3	0.1	1/3	0.4	0.9016	0.8973	1.0696	1.0789
		0.3	0.1	1	0	0.8325	0.8396	1.0126	1.0161
		0.3	0.1	1	0.4	1.0092	1.0152	1.2012	1.2203
		0.1	0.3	1/3	0	1.0092	1.0081	1.1512	1.1660
		0.1	0.3	1/3	0.4	1.1723	1.1819	1.3166	1.3343
		0.1	0.3	1	0	1.0973	1.1107	1.2663	1.2920
		0.1	0.3	1	0.4	1.2652	1.2891	1.4257	1.4632

Table S6. W_{mean}'s of the BNM, BUM, BNP and BUP methods under $(p_f, p_m) = (0.3, 0.1)$ and $(0.1, 0.3)$, and $(\sigma_{e0}^2, \sigma_{e1}^2, \sigma_{e2}^2) = (1, 1, 1)$ among 500 replicates for mixed data and only general pedigrees, respectively.

Trait	(N_p, n_{If})	p_f	p_m	σ_g^2	MR	Mixed Data		Pedigrees	
						BNM	BUM	BNP	BUP
Quantitative	(150, 650)	0.3	0.1	1/3	0	0.9741	0.9803	1.2622	1.2779
		0.3	0.1	1/3	0.4	1.1531	1.1628	1.4089	1.4319
		0.3	0.1	1	0	1.1256	1.1368	1.3718	1.3938
		0.3	0.1	1	0.4	1.2984	1.3158	1.5058	1.5336
		0.1	0.3	1/3	0	1.3153	1.3362	1.4432	1.4712
		0.1	0.3	1/3	0.4	1.4302	1.4538	1.5299	1.5614
		0.1	0.3	1	0	1.3882	1.4108	1.5216	1.5538
		0.1	0.3	1	0.4	1.5008	1.5319	1.6075	1.6440
	(600, 2600)	0.3	0.1	1/3	0	0.5812	0.5844	0.7734	0.7772
		0.3	0.1	1/3	0.4	0.7238	0.7286	0.9407	0.9440
		0.3	0.1	1	0	0.6612	0.6628	0.8976	0.9034
		0.3	0.1	1	0.4	0.8211	0.8224	1.0689	1.0734
		0.1	0.3	1/3	0	0.7940	0.7994	1.0271	1.0370
		0.1	0.3	1/3	0.4	0.9620	0.9671	1.1812	1.1949
		0.1	0.3	1	0	0.9161	0.9247	1.1441	1.1577
		0.1	0.3	1	0.4	1.0997	1.1076	1.3007	1.3158
Qualitative	(150, 650)	0.3	0.1	1/3	0	1.1441	1.1519	1.4151	1.4366
		0.3	0.1	1/3	0.4	1.3134	1.3321	1.5496	1.5837
		0.3	0.1	1	0	1.2984	1.3100	1.5248	1.5528
		0.3	0.1	1	0.4	1.4367	1.4629	1.6093	1.6455
		0.1	0.3	1/3	0	1.3287	1.3428	1.5318	1.5645
		0.1	0.3	1/3	0.4	1.4466	1.4723	1.6001	1.6344
		0.1	0.3	1	0	1.4432	1.4671	1.6046	1.6383
		0.1	0.3	1	0.4	1.5272	1.5578	1.6388	1.6737
	(600, 2600)	0.3	0.1	1/3	0	0.7232	0.7261	0.8966	0.9015
		0.3	0.1	1/3	0.4	0.8885	0.8911	1.0646	1.0733
		0.3	0.1	1	0	0.8360	0.8403	1.0265	1.0326
		0.3	0.1	1	0.4	1.0182	1.0238	1.2101	1.2234
		0.1	0.3	1/3	0	0.9689	0.9733	1.1336	1.1431
		0.1	0.3	1/3	0.4	1.1400	1.1475	1.2879	1.3007
		0.1	0.3	1	0	1.0978	1.1077	1.2717	1.2881
		0.1	0.3	1	0.4	1.2446	1.2590	1.3986	1.4204

Table S7. W_{iqr}'s of the BNM, BUM, BNP and BUP methods under $(p_f, p_m) = (0.3, 0.1)$ and $(0.1, 0.3)$, and $(\sigma_{e0}^2, \sigma_{e1}^2, \sigma_{e2}^2) = (1, 1, 1)$ among 500 replicates for mixed data and only general pedigrees, respectively.

Trait	(N_p, n_{If})	p_f	p_m	σ_g^2	MR	Mixed Data		Pedigrees	
						BNM	BUM	BNP	BUP
Quantitative	(150, 650)	0.3	0.1	1/3	0	0.3617	0.3985	0.4307	0.5029
		0.3	0.1	1/3	0.4	0.4538	0.4987	0.4214	0.4770
		0.3	0.1	1	0	0.4622	0.5181	0.4585	0.5193
		0.3	0.1	1	0.4	0.4590	0.5321	0.3923	0.4226
		0.1	0.3	1/3	0	0.3802	0.4455	0.3684	0.4124
		0.1	0.3	1/3	0.4	0.3668	0.4195	0.3616	0.3711
		0.1	0.3	1	0	0.4164	0.4700	0.3560	0.3938
		0.1	0.3	1	0.4	0.3440	0.3973	0.2597	0.2627
	(600, 2600)	0.3	0.1	1/3	0	0.1943	0.2092	0.2664	0.3142
		0.3	0.1	1/3	0.4	0.2732	0.3050	0.3496	0.3929
		0.3	0.1	1	0	0.2178	0.2526	0.3543	0.4123
		0.3	0.1	1	0.4	0.3023	0.3558	0.4058	0.4629
		0.1	0.3	1/3	0	0.3054	0.3419	0.3489	0.4103
		0.1	0.3	1/3	0.4	0.3862	0.4262	0.4167	0.5082
		0.1	0.3	1	0	0.3291	0.3803	0.3984	0.4507
		0.1	0.3	1	0.4	0.3352	0.3875	0.4101	0.4746
Qualitative	(150, 650)	0.3	0.1	1/3	0	0.4155	0.4763	0.4602	0.5238
		0.3	0.1	1/3	0.4	0.4345	0.5088	0.3481	0.3742
		0.3	0.1	1	0	0.5088	0.5707	0.3967	0.4345
		0.3	0.1	1	0.4	0.4309	0.4966	0.2982	0.3034
		0.1	0.3	1/3	0	0.4188	0.4645	0.3671	0.3828
		0.1	0.3	1/3	0.4	0.4170	0.4648	0.2751	0.2921
		0.1	0.3	1	0	0.3815	0.4362	0.2900	0.3043
		0.1	0.3	1	0.4	0.3332	0.3724	0.2301	0.2311
	(600, 2600)	0.3	0.1	1/3	0	0.2425	0.2740	0.3214	0.3587
		0.3	0.1	1/3	0.4	0.3283	0.3828	0.4143	0.4652
		0.3	0.1	1	0	0.3163	0.3584	0.4030	0.4552
		0.3	0.1	1	0.4	0.3941	0.4397	0.4512	0.5212
		0.1	0.3	1/3	0	0.3805	0.4372	0.4075	0.4719
		0.1	0.3	1/3	0.4	0.4038	0.4658	0.4539	0.5111
		0.1	0.3	1	0	0.4024	0.4724	0.4329	0.4947
		0.1	0.3	1	0.4	0.4351	0.4800	0.4139	0.4686

Table S8. W_{sd}'s of the BNM, BUM, BNP and BUP methods under $(p_f, p_m) = (0.3, 0.1)$ and $(0.1, 0.3)$, and $(\sigma_{e0}^2, \sigma_{e1}^2, \sigma_{e2}^2) = (1, 1, 1)$ among 500 replicates for mixed data and only general pedigrees, respectively.

Trait	(N_p, n_{If})	p_f	p_m	σ_g^2	MR	Mixed Data		Pedigrees	
						BNM	BUM	BNP	BUP
Quantitative	(150, 650)	0.3	0.1	1/3	0	0.2710	0.2991	0.2971	0.3307
		0.3	0.1	1/3	0.4	0.3038	0.3356	0.2874	0.3174
		0.3	0.1	1	0	0.3052	0.3359	0.2965	0.3291
		0.3	0.1	1	0.4	0.3050	0.3386	0.2620	0.2883
		0.1	0.3	1/3	0	0.2819	0.3093	0.2563	0.2806
		0.1	0.3	1/3	0.4	0.2553	0.2841	0.2525	0.2761
		0.1	0.3	1	0	0.2873	0.3172	0.2557	0.2838
		0.1	0.3	1	0.4	0.2489	0.2773	0.2050	0.2282
	(600, 2600)	0.3	0.1	1/3	0	0.1528	0.1630	0.2137	0.2334
		0.3	0.1	1/3	0.4	0.2137	0.2275	0.2578	0.2822
		0.3	0.1	1	0	0.1799	0.1925	0.2720	0.2966
		0.3	0.1	1	0.4	0.2397	0.2599	0.3063	0.3349
		0.1	0.3	1/3	0	0.2440	0.2638	0.2885	0.3140
		0.1	0.3	1/3	0.4	0.2833	0.3071	0.3072	0.3383
		0.1	0.3	1	0	0.2644	0.2859	0.3001	0.3295
		0.1	0.3	1	0.4	0.2859	0.3117	0.2916	0.3234
Qualitative	(150, 650)	0.3	0.1	1/3	0	0.3021	0.3334	0.2918	0.3239
		0.3	0.1	1/3	0.4	0.3108	0.3427	0.2585	0.2873
		0.3	0.1	1	0	0.3253	0.3573	0.2814	0.3089
		0.3	0.1	1	0.4	0.3005	0.3346	0.2360	0.2571
		0.1	0.3	1/3	0	0.3034	0.3337	0.2531	0.2796
		0.1	0.3	1/3	0.4	0.2930	0.3219	0.2366	0.2616
		0.1	0.3	1	0	0.2662	0.2950	0.2233	0.2432
		0.1	0.3	1	0.4	0.2423	0.2683	0.2095	0.2344
	(600, 2600)	0.3	0.1	1/3	0	0.1905	0.2061	0.2523	0.2743
		0.3	0.1	1/3	0.4	0.2497	0.2729	0.3013	0.3293
		0.3	0.1	1	0	0.2328	0.2573	0.2846	0.3138
		0.3	0.1	1	0.4	0.2838	0.3114	0.3083	0.3400
		0.1	0.3	1/3	0	0.2939	0.3203	0.3121	0.3429
		0.1	0.3	1/3	0.4	0.3251	0.3557	0.3262	0.3623
		0.1	0.3	1	0	0.3073	0.3404	0.3093	0.3376
		0.1	0.3	1	0.4	0.3041	0.3356	0.2835	0.3152

Table S9. Mean squared errors (MSEs) of point estimates $\hat{\gamma}_{BNM}$, $\hat{\gamma}_{BUM}$, $\hat{\gamma}_{BNP}$ and $\hat{\gamma}_{BUP}$ under $p_f = p_m$, and $(\sigma_{e0}^2, \sigma_{e1}^2, \sigma_{e2}^2) = (1, 1, 1)$ and $(1, 1.2, 1)$ among 500 replicates for quantitative traits.

(N_p, n_{If})	p_f	σ_g^2	MR	$(\sigma_{e0}^2, \sigma_{e1}^2, \sigma_{e2}^2) = (1, 1, 1)$				$(\sigma_{e0}^2, \sigma_{e1}^2, \sigma_{e2}^2) = (1, 1.2, 1)$			
				$\hat{\gamma}_{BNM}$	$\hat{\gamma}_{BUM}$	$\hat{\gamma}_{BNP}$	$\hat{\gamma}_{BUP}$	$\hat{\gamma}_{BNM}$	$\hat{\gamma}_{BUM}$	$\hat{\gamma}_{BNP}$	$\hat{\gamma}_{BUP}$
(150, 650)	0.3	1/3	0	0.0643	0.0707	0.1167	0.1342	0.0650	0.0710	0.1192	0.1278
	0.3	1/3	0.4	0.0943	0.1032	0.1564	0.1757	0.0902	0.1027	0.1600	0.1770
	0.3	1	0	0.0889	0.0966	0.1528	0.1646	0.0874	0.0881	0.1459	0.1523
	0.3	1	0.4	0.1323	0.1473	0.2086	0.2409	0.1358	0.1363	0.1993	0.2192
	0.1	1/3	0	0.1850	0.1968	0.2959	0.3247	0.1846	0.1902	0.3071	0.3282
	0.1	1/3	0.4	0.2455	0.2670	0.3781	0.4325	0.2355	0.2515	0.3779	0.4332
	0.1	1	0	0.2010	0.2192	0.3399	0.3792	0.2103	0.2168	0.3207	0.3653
	0.1	1	0.4	0.2754	0.3064	0.4224	0.4900	0.2821	0.3064	0.4399	0.5052
(600, 2600)	0.3	1/3	0	0.0229	0.0236	0.0407	0.0421	0.0219	0.0227	0.0443	0.0462
	0.3	1/3	0.4	0.0359	0.0377	0.0570	0.0606	0.0310	0.0323	0.0596	0.0623
	0.3	1	0	0.0256	0.0260	0.0543	0.0576	0.0264	0.0267	0.0556	0.0578
	0.3	1	0.4	0.0416	0.0445	0.0805	0.0879	0.0427	0.0445	0.0768	0.0821
	0.1	1/3	0	0.0786	0.0846	0.1210	0.1300	0.0811	0.0837	0.1256	0.1346
	0.1	1/3	0.4	0.1147	0.1205	0.1689	0.1750	0.1206	0.1251	0.1758	0.1846
	0.1	1	0	0.0962	0.1039	0.1650	0.1773	0.0873	0.0970	0.1614	0.1706
	0.1	1	0.4	0.1353	0.1415	0.2080	0.2252	0.1292	0.1407	0.2025	0.2172

Table S10. Coverage probability (CPs, in %) of the BNM, BUM, BNP and BUP methods under $p_f = p_m$, and $(\sigma_{e0}^2, \sigma_{e1}^2, \sigma_{e2}^2) = (1, 1, 1)$ and $(1, 1.2, 1)$ among 500 replicates for quantitative traits [a].

(N_p, n_{If})	p_f	σ_g^2	MR	$(\sigma_{e0}^2, \sigma_{e1}^2, \sigma_{e2}^2) = (1, 1, 1)$				$(\sigma_{e0}^2, \sigma_{e1}^2, \sigma_{e2}^2) = (1, 1.2, 1)$			
				**BNM**	**BUM**	**BNP**	**BUP**	**BNM**	**BUM**	**BNP**	**BUP**
(150, 650)	0.3	1/3	0	94.6	96.2	95.4	96.2	94.8	96.0	95.4	96.0
	0.3	1/3	0.4	95.0	95.6	95.2	95.6	95.0	95.4	95.4	95.6
	0.3	1	0	95.0	95.0	93.8	94.6	94.8	95.0	93.8	94.8
	0.3	1	0.4	94.4	95.2	94.2	95.0	94.4	95.4	94.4	95.0
	0.1	1/3	0	93.8	93.6	95.8	94.4	94.0	93.8	95.6	94.4
	0.1	1/3	0.4	93.4	95.4	95.0	94.2	93.6	95.2	95.0	94.2
	0.1	1	0	96.2	94.6	94.0	95.0	96.0	94.4	94.4	95.0
	0.1	1	0.4	94.4	95.8	94.2	95.4	94.6	95.8	94.2	95.2
(600, 2600)	0.3	1/3	0	94.2	94.2	94.0	94.4	94.2	94.4	94.2	94.4
	0.3	1/3	0.4	94.2	94.4	95.6	95.4	94.0	94.4	95.4	95.4
	0.3	1	0	95.0	96.4	95.6	95.8	95.0	96.2	95.6	95.8
	0.3	1	0.4	95.8	95.8	94.6	94.6	95.6	95.8	94.8	94.2
	0.1	1/3	0	94.2	94.2	95.0	94.6	94.4	94.4	95.0	94.6
	0.1	1/3	0.4	94.0	95.0	94.6	95.2	94.2	95.0	94.6	95.2
	0.1	1	0	95.6	95.6	95.6	96.0	95.6	95.4	95.4	96.2
	0.1	1	0.4	95.0	95.4	94.8	95.6	95.2	95.6	94.8	95.6

[a] The empirical CP should be between 93.05% and 96.95% $(0.95 \pm 2 \times \sqrt{\frac{0.95\times0.05}{500}})$ with 95% probability.

Table S11. W_{median}'s of the BNM, BUM, BNP and BUP methods under $p_f = p_m$, and $(\sigma_{e0}^2, \sigma_{e1}^2, \sigma_{e2}^2) = (1, 1, 1)$ and $(1, 1.2, 1)$ among 500 replicates for quantitative traits.

(N_p, n_{If})	p_f	σ_g^2	MR	$(\sigma_{e0}^2, \sigma_{e1}^2, \sigma_{e2}^2) = (1, 1, 1)$				$(\sigma_{e0}^2, \sigma_{e1}^2, \sigma_{e2}^2) = (1, 1.2, 1)$			
				**BNM**	**BUM**	**BNP**	**BUP**	**BNM**	**BUM**	**BNP**	**BUP**
(150, 650)	0.3	1/3	0	0.9770	0.9815	1.2152	1.2336	0.9664	0.9789	1.2278	1.2426
	0.3	1/3	0.4	1.1601	1.1801	1.4467	1.4781	1.1659	1.1725	1.4732	1.5127
	0.3	1	0	1.0627	1.0636	1.3667	1.3966	1.0590	1.0622	1.3564	1.4011
	0.3	1	0.4	1.2572	1.2627	1.5525	1.6017	1.2601	1.2737	1.5762	1.6176
	0.1	1/3	0	1.4258	1.4452	1.5863	1.6305	1.4129	1.4533	1.5911	1.6471
	0.1	1/3	0.4	1.5502	1.5935	1.6720	1.7201	1.5485	1.5970	1.6821	1.7291
	0.1	1	0	1.5453	1.5940	1.6713	1.7166	1.5562	1.6082	1.6696	1.7122
	0.1	1	0.4	1.6493	1.6999	1.7109	1.7633	1.6353	1.6908	1.7077	1.7591
(600, 2600)	0.3	1/3	0	0.5350	0.5378	0.7332	0.7324	0.5428	0.5481	0.7227	0.7334
	0.3	1/3	0.4	0.6658	0.6642	0.8894	0.8840	0.6726	0.6749	0.8756	0.8859
	0.3	1	0	0.6216	0.6272	0.8754	0.8861	0.6306	0.6308	0.8776	0.8760
	0.3	1	0.4	0.7755	0.7868	1.0389	1.0515	0.7689	0.7650	0.9363	0.9294
	0.1	1/3	0	1.0073	1.0217	1.2340	1.2505	1.0081	1.0209	1.2200	1.2400
	0.1	1/3	0.4	1.1494	1.1790	1.3633	1.3885	1.1597	1.1879	1.3542	1.3752
	0.1	1	0	1.1208	1.1298	1.3468	1.3654	1.1301	1.1355	1.3324	1.3507
	0.1	1	0.4	1.3067	1.3313	1.4664	1.5054	1.2963	1.3238	1.4426	1.4902

Table S12. W_{mean}'s of the BNM, BUM, BNP and BUP methods under $p_f = p_m$, and $(\sigma_{e0}^2, \sigma_{e1}^2, \sigma_{e2}^2) = (1, 1, 1)$ and $(1, 1.2, 1)$ among 500 replicates for quantitative traits.

(N_p, n_{If})	p_f	σ_g^2	MR	$(\sigma_{e0}^2, \sigma_{e1}^2, \sigma_{e2}^2) = (1, 1, 1)$				$(\sigma_{e0}^2, \sigma_{e1}^2, \sigma_{e2}^2) = (1, 1.2, 1)$			
				**BNM**	**BUM**	**BNP**	**BUP**	**BNM**	**BUM**	**BNP**	**BUP**
(150, 650)	0.3	1/3	0	0.9751	0.9813	1.2201	1.2367	0.9756	0.9825	1.2334	1.2495
	0.3	1/3	0.4	1.1625	1.1727	1.3898	1.4133	1.1624	1.1705	1.3967	1.4220
	0.3	1	0	1.0614	1.0689	1.3336	1.3570	1.0683	1.0779	1.3406	1.3631
	0.3	1	0.4	1.2494	1.2615	1.4847	1.5154	1.2566	1.2736	1.4968	1.5201
	0.1	1/3	0	1.3950	1.4138	1.5357	1.5659	1.3880	1.4030	1.5300	1.5636
	0.1	1/3	0.4	1.5004	1.5320	1.6064	1.6438	1.4960	1.5264	1.6110	1.6564
	0.1	1	0	1.5014	1.5308	1.6002	1.6392	1.5021	1.5312	1.5906	1.6291
	0.1	1	0.4	1.5957	1.6336	1.6634	1.7040	1.5971	1.6242	1.6755	1.7032
(600, 2600)	0.3	1/3	0	0.5355	0.5359	0.7217	0.7242	0.5305	0.5332	0.7246	0.7288
	0.3	1/3	0.4	0.6649	0.6678	0.8832	0.8856	0.6677	0.6715	0.8881	0.8920
	0.3	1	0	0.6209	0.6250	0.8600	0.8676	0.6197	0.6196	0.8619	0.8741
	0.3	1	0.4	0.7758	0.7836	1.0461	1.0538	0.7642	0.7855	1.0549	1.0665
	0.1	1/3	0	1.0084	1.0166	1.2252	1.2418	1.0130	1.0225	1.2254	1.2594
	0.1	1/3	0.4	1.1690	1.1854	1.3583	1.3799	1.1702	1.1911	1.3402	1.3799
	0.1	1	0	1.1193	1.1312	1.3362	1.3541	1.1247	1.1457	1.3347	1.3637
	0.1	1	0.4	1.2850	1.3036	1.4442	1.4686	1.2760	1.2928	1.4479	1.4610

Table S13. W_{iqr}'s of the BNM, BUM, BNP and BUP methods under $p_f = p_m$, and $(\sigma_{e0}^2, \sigma_{e1}^2, \sigma_{e2}^2) = (1, 1, 1)$ and $(1, 1.2, 1)$ among 500 replicates for quantitative traits.

(N_p, n_{If})	p_f	σ_g^2	MR	$(\sigma_{e0}^2, \sigma_{e1}^2, \sigma_{e2}^2) = (1, 1, 1)$				$(\sigma_{e0}^2, \sigma_{e1}^2, \sigma_{e2}^2) = (1, 1.2, 1)$			
				**BNM**	**BUM**	**BNP**	**BUP**	**BNM**	**BUM**	**BNP**	**BUP**
(150, 650)	0.3	1/3	0	0.3333	0.3700	0.4620	0.5268	0.3421	0.3846	0.4631	0.5275
	0.3	1/3	0.4	0.4106	0.4680	0.4760	0.5466	0.4192	0.4715	0.4652	0.5343
	0.3	1	0	0.4218	0.4755	0.4739	0.5418	0.4127	0.4635	0.4628	0.5396
	0.3	1	0.4	0.4351	0.5176	0.4162	0.4673	0.4382	0.5029	0.4006	0.4537
	0.1	1/3	0	0.4058	0.4620	0.3309	0.3603	0.3930	0.4453	0.3292	0.3591
	0.1	1/3	0.4	0.3389	0.3812	0.2603	0.2690	0.3246	0.3765	0.2504	0.2647
	0.1	1	0	0.3345	0.3817	0.2612	0.2806	0.3468	0.3919	0.2727	0.2925
	0.1	1	0.4	0.2467	0.2585	0.1618	0.1658	0.2523	0.2653	0.1715	0.1705
(600, 2600)	0.3	1/3	0	0.1590	0.1629	0.2391	0.2655	0.1570	0.1640	0.2138	0.2433
	0.3	1/3	0.4	0.2184	0.2344	0.3295	0.3803	0.2292	0.2444	0.3140	0.3779
	0.3	1	0	0.1946	0.2087	0.3241	0.3795	0.2057	0.2147	0.3158	0.3634
	0.3	1	0.4	0.2733	0.3080	0.3892	0.4434	0.2795	0.3052	0.3757	0.4363
	0.1	1/3	0	0.3785	0.4380	0.3878	0.4345	0.3590	0.4201	0.3754	0.4251
	0.1	1/3	0.4	0.4386	0.4744	0.3998	0.4649	0.4257	0.4631	0.3847	0.4532
	0.1	1	0	0.3744	0.4297	0.4101	0.4489	0.3849	0.4358	0.4090	0.4450
	0.1	1	0.4	0.3333	0.3700	0.3640	0.4160	0.3451	0.3802	0.3723	0.4253

Table S14. W_{sd}'s of the BNM, BUM, BNP and BUP methods under $p_f = p_m$, and $(\sigma_{e0}^2, \sigma_{e1}^2, \sigma_{e2}^2) = (1, 1, 1)$ and $(1, 1.2, 1)$ among 500 replicates for quantitative traits.

(N_p, n_{If})	p_f	σ_g^2	MR	$(\sigma_{e0}^2, \sigma_{e1}^2, \sigma_{e2}^2) = (1, 1, 1)$				$(\sigma_{e0}^2, \sigma_{e1}^2, \sigma_{e2}^2) = (1, 1.2, 1)$			
				**BNM**	**BUM**	**BNP**	**BUP**	**BNM**	**BUM**	**BNP**	**BUP**
(150, 650)	0.3	1/3	0	0.2568	0.2811	0.3067	0.3426	0.2597	0.2863	0.3010	0.3475
	0.3	1/3	0.4	0.2946	0.3250	0.3100	0.3403	0.3028	0.3313	0.3136	0.3349
	0.3	1	0	0.2926	0.3231	0.3180	0.3523	0.2983	0.3214	0.3039	0.3556
	0.3	1	0.4	0.3099	0.3432	0.2815	0.3107	0.3094	0.3319	0.2874	0.3169
	0.1	1/3	0	0.2797	0.3112	0.2366	0.2614	0.2894	0.3166	0.2485	0.2745
	0.1	1/3	0.4	0.2542	0.2825	0.2179	0.2442	0.2569	0.2725	0.2130	0.2477
	0.1	1	0	0.2309	0.2603	0.2136	0.2353	0.2445	0.2605	0.2125	0.2342
	0.1	1	0.4	0.1903	0.2110	0.1758	0.1935	0.1970	0.2101	0.1828	0.1947
(600, 2600)	0.3	1/3	0	0.1229	0.1284	0.1827	0.1985	0.1206	0.1278	0.1798	0.1960
	0.3	1/3	0.4	0.1669	0.1793	0.2385	0.2643	0.1773	0.1808	0.2374	0.2590
	0.3	1	0	0.1558	0.1665	0.2412	0.2665	0.1585	0.1686	0.2490	0.2595
	0.3	1	0.4	0.2126	0.2317	0.2866	0.3172	0.2076	0.2238	0.2754	0.3197
	0.1	1/3	0	0.2979	0.3230	0.2909	0.3178	0.2988	0.3240	0.2867	0.3178
	0.1	1/3	0.4	0.3013	0.3298	0.2775	0.3045	0.3095	0.3257	0.2830	0.3021
	0.1	1	0	0.2980	0.3280	0.2918	0.3206	0.2959	0.3260	0.2896	0.3200
	0.1	1	0.4	0.2845	0.3104	0.2576	0.2866	0.2779	0.3161	0.2584	0.2872

Table S15. Type I error rate of our proposed improved linear mixed model under $p_f = p_m$ and $(\sigma_{e0}^2, \sigma_{e1}^2, \sigma_{e2}^2) = (1, 1, 1)$ among 1000 replicates.

$(\boldsymbol{N_p}, \boldsymbol{n_{If}})$	$\boldsymbol{p_f}$	$\boldsymbol{\sigma_g^2}$	**Type I Error Rate**
(150, 650)	0.3	1/3	0.046
	0.3	1	0.053
	0.1	1/3	0.051
	0.1	1	0.043
(600, 2600)	0.3	1/3	0.049
	0.3	1	0.055
	0.1	1/3	0.046
	0.1	1	0.053

Supplementary Figures

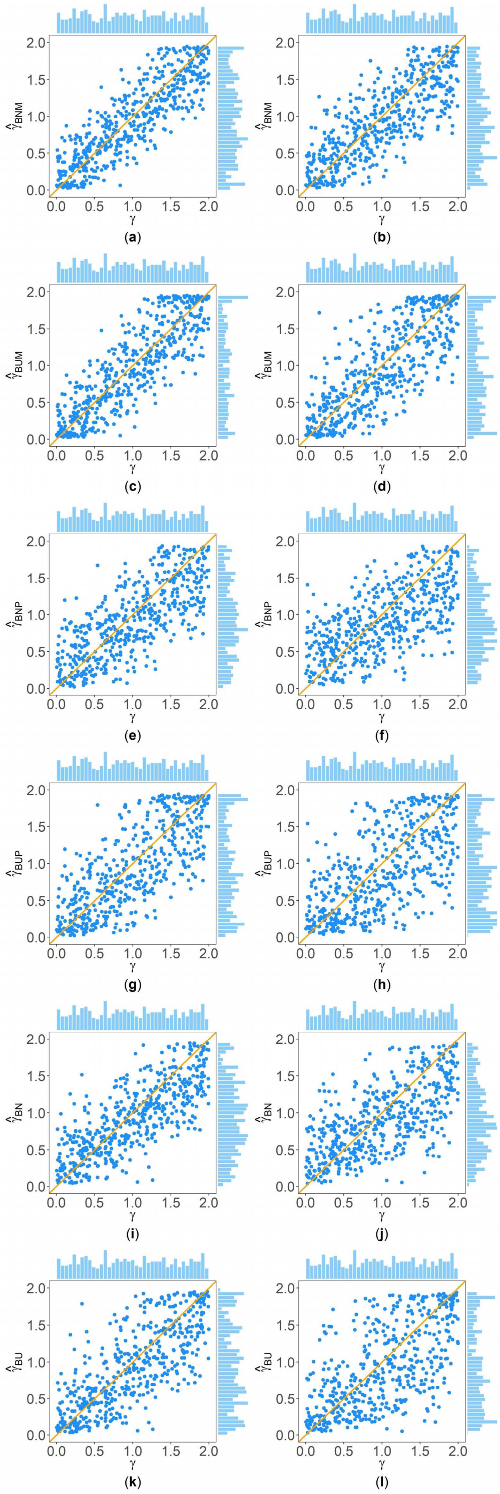

Figure S1. Scatter plots of six point estimates of γ against true values of γ with $N_p = 150$, $n_{If} = 650$, $p_f = p_m = 0.3$, $\sigma_g^2 = 1$, $(\sigma_{e0}^2, \sigma_{e1}^2, \sigma_{e2}^2) = (1, 1, 1)$ and $MR = \{0, 0.4\}$ for quantitative trait. The upper side and the right side of each subplot are respectively the distribution of the true value of γ and that of the point estimate of γ. (**a**) $\hat{\gamma}_{BNM}$ with $MR = 0$; (**b**) $\hat{\gamma}_{BNM}$ with $MR = 0.4$; (**c**) $\hat{\gamma}_{BUM}$ with $MR = 0$; (**d**) $\hat{\gamma}_{BUM}$ with $MR = 0.4$; (**e**) $\hat{\gamma}_{BNP}$ with $MR = 0$; (**f**) $\hat{\gamma}_{BNP}$ with $MR = 0.4$; (**g**) $\hat{\gamma}_{BUP}$ with $MR = 0$; (**h**) $\hat{\gamma}_{BUP}$ with $MR = 0.4$; (**i**) $\hat{\gamma}_{BN}$ with $MR = 0$; (**j**) $\hat{\gamma}_{BN}$ with $MR = 0.4$; (**k**) $\hat{\gamma}_{BU}$ with $MR = 0$; (**l**) $\hat{\gamma}_{BU}$ with $MR = 0.4$.

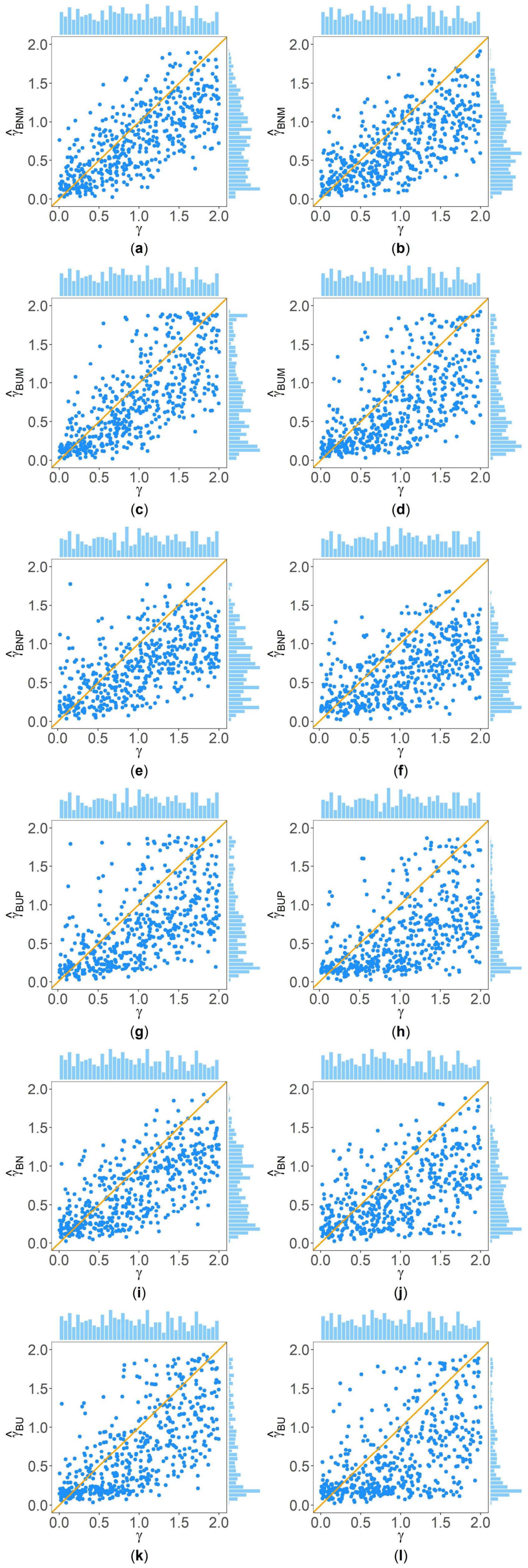

Figure S2. Scatter plots of six point estimates of γ against true values of γ with $N_p = 150$, $n_{If} = 650$, $p_f = p_m = 0.1$, $\sigma_g^2 = 1/3$, $(\sigma_{e0}^2, \sigma_{e1}^2, \sigma_{e2}^2) = (1,1,1)$ and $MR = \{0, 0.4\}$ for quantitative trait. The upper side and the right side of each subplot are respectively the distribution of the true value of γ and that of the point estimate of γ. (**a**) $\hat{\gamma}_{BNM}$ with $MR = 0$; (**b**) $\hat{\gamma}_{BNM}$ with $MR = 0.4$; (**c**) $\hat{\gamma}_{BUM}$ with $MR = 0$; (**d**) $\hat{\gamma}_{BUM}$ with $MR = 0.4$; (**e**) $\hat{\gamma}_{BNP}$ with $MR = 0$; (**f**) $\hat{\gamma}_{BNP}$ with $MR = 0.4$; (**g**) $\hat{\gamma}_{BUP}$ with $MR = 0$; (**h**) $\hat{\gamma}_{BUP}$ with $MR = 0.4$; (**i**) $\hat{\gamma}_{BN}$ with $MR = 0$; (**j**) $\hat{\gamma}_{BN}$ with $MR = 0.4$; (**k**) $\hat{\gamma}_{BU}$ with $MR = 0$; (**l**) $\hat{\gamma}_{BU}$ with $MR = 0.4$.

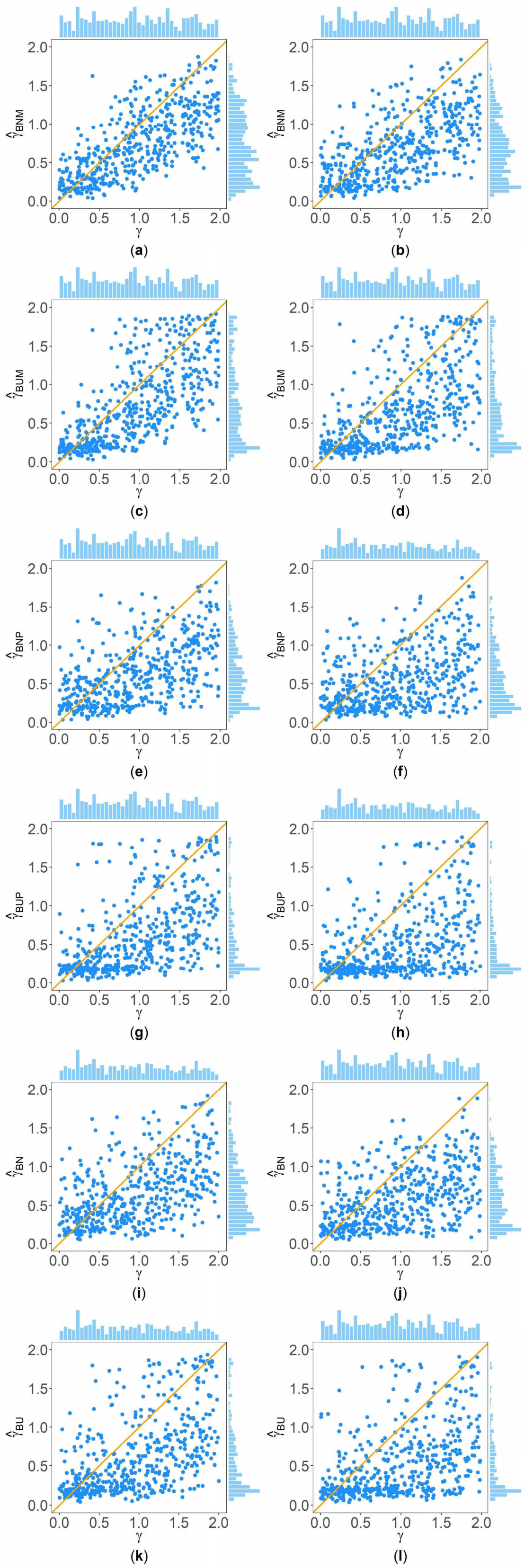

Figure S3. Scatter plots of six point estimates of γ against true values of γ with $N_p = 150$, $n_{If} = 650$, $p_f = p_m = 0.1$, $\sigma_g^2 = 1$, $(\sigma_{e0}^2, \sigma_{e1}^2, \sigma_{e2}^2) = (1, 1, 1)$ and $MR = \{0, 0.4\}$ for quantitative trait. The upper side and the right side of each subplot are respectively the distribution of the true value of γ and that of the point estimate of γ. (**a**) $\hat{\gamma}_{BNM}$ with $MR = 0$; (**b**) $\hat{\gamma}_{BNM}$ with $MR = 0.4$; (**c**) $\hat{\gamma}_{BUM}$ with $MR = 0$; (**d**) $\hat{\gamma}_{BUM}$ with $MR = 0.4$; (**e**) $\hat{\gamma}_{BNP}$ with $MR = 0$; (**f**) $\hat{\gamma}_{BNP}$ with $MR = 0.4$; (**g**) $\hat{\gamma}_{BUP}$ with $MR = 0$; (**h**) $\hat{\gamma}_{BUP}$ with $MR = 0.4$; (**i**) $\hat{\gamma}_{BN}$ with $MR = 0$; (**j**) $\hat{\gamma}_{BN}$ with $MR = 0.4$; (**k**) $\hat{\gamma}_{BU}$ with $MR = 0$; (**l**) $\hat{\gamma}_{BU}$ with $MR = 0.4$.

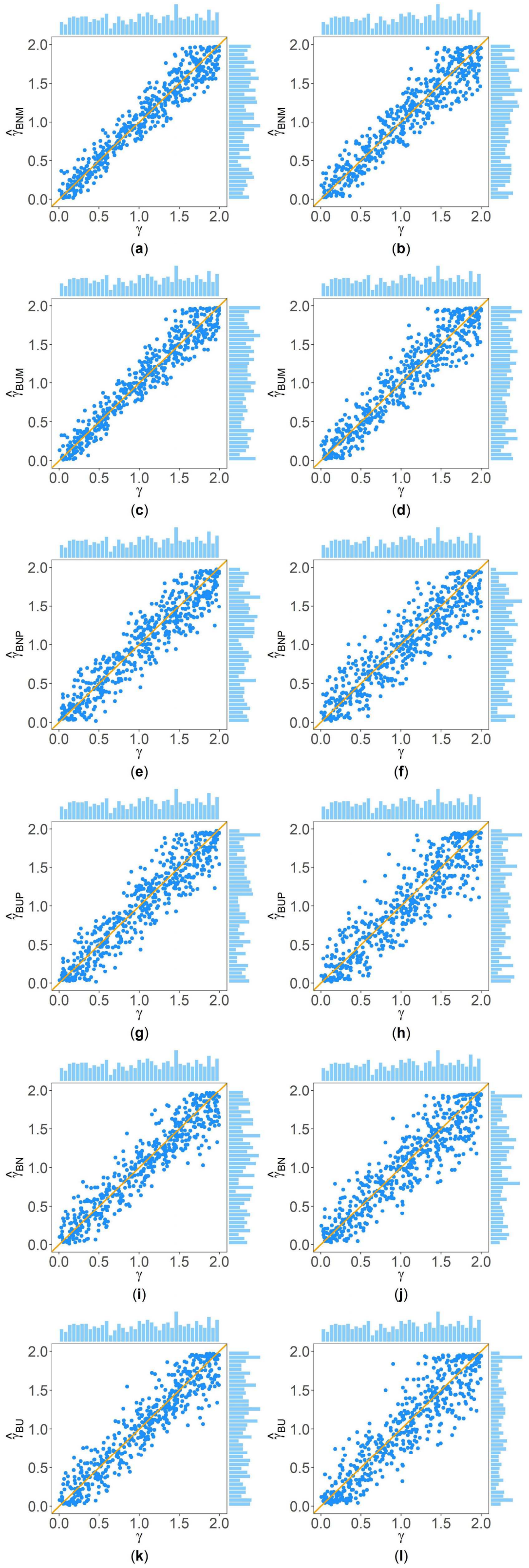

Figure S4. Scatter plots of six point estimates of γ against true values of γ with $N_p = 600$, $n_{If} = 2600$, $p_f = p_m = 0.3$, $\sigma_g^2 = 1/3$, $(\sigma_{e0}^2, \sigma_{e1}^2, \sigma_{e2}^2) = (1, 1, 1)$ and $MR = \{0, 0.4\}$ for quantitative trait. The upper side and the right side of each subplot are respectively the distribution of the true value of γ and that of the point estimate of γ. (**a**) $\hat{\gamma}_{BNM}$ with $MR = 0$; (**b**) $\hat{\gamma}_{BNM}$ with $MR = 0.4$; (**c**) $\hat{\gamma}_{BUM}$ with $MR = 0$; (**d**) $\hat{\gamma}_{BUM}$ with $MR = 0.4$; (**e**) $\hat{\gamma}_{BNP}$ with $MR = 0$; (**f**) $\hat{\gamma}_{BNP}$ with $MR = 0.4$; (**g**) $\hat{\gamma}_{BUP}$ with $MR = 0$; (**h**) $\hat{\gamma}_{BUP}$ with $MR = 0.4$; (**i**) $\hat{\gamma}_{BN}$ with $MR = 0$; (**j**) $\hat{\gamma}_{BN}$ with $MR = 0.4$; (**k**) $\hat{\gamma}_{BU}$ with $MR = 0$; (**l**) $\hat{\gamma}_{BU}$ with $MR = 0.4$.

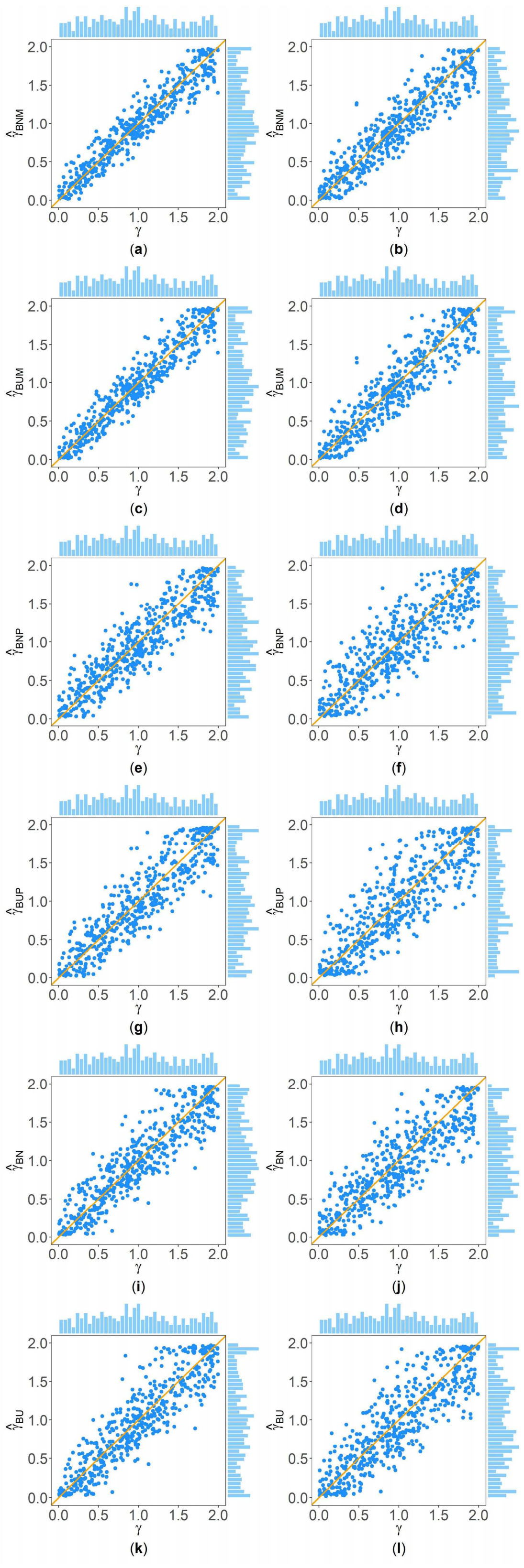

Figure S5. Scatter plots of six point estimates of γ against true values of γ with $N_p = 600$, $n_{If} = 2600$, $p_f = p_m = 0.3$, $\sigma_g^2 = 1$, $(\sigma_{e0}^2, \sigma_{e1}^2, \sigma_{e2}^2) = (1, 1, 1)$ and $MR = \{0, 0.4\}$ for quantitative trait. The upper side and the right side of each subplot are respectively the distribution of the true value of γ and that of the point estimate of γ. (**a**) $\hat{\gamma}_{BNM}$ with $MR = 0$; (**b**) $\hat{\gamma}_{BNM}$ with $MR = 0.4$; (**c**) $\hat{\gamma}_{BUM}$ with $MR = 0$; (**d**) $\hat{\gamma}_{BUM}$ with $MR = 0.4$; (**e**) $\hat{\gamma}_{BNP}$ with $MR = 0$; (**f**) $\hat{\gamma}_{BNP}$ with $MR = 0.4$; (**g**) $\hat{\gamma}_{BUP}$ with $MR = 0$; (**h**) $\hat{\gamma}_{BUP}$ with $MR = 0.4$; (**i**) $\hat{\gamma}_{BN}$ with $MR = 0$; (**j**) $\hat{\gamma}_{BN}$ with $MR = 0.4$; (**k**) $\hat{\gamma}_{BU}$ with $MR = 0$; (**l**) $\hat{\gamma}_{BU}$ with $MR = 0.4$.

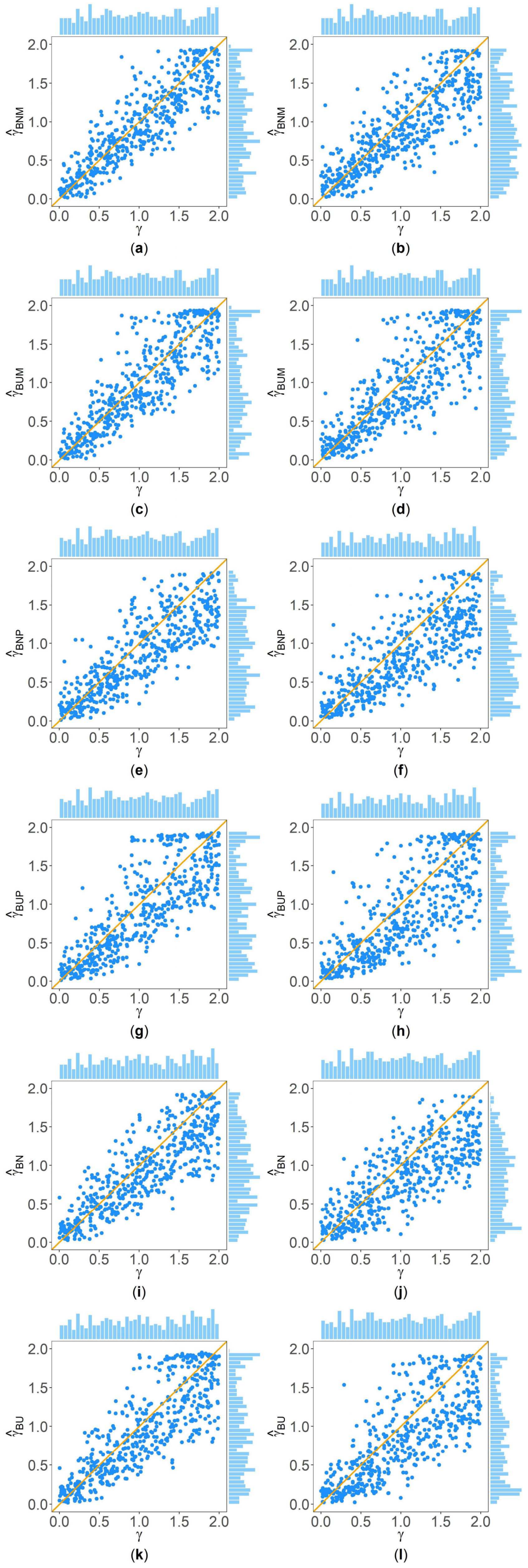

Figure S6. Scatter plots of six point estimates of γ against true values of γ with $N_p = 600$, $n_{If} = 2600$, $p_f = p_m = 0.1$, $\sigma_g^2 = 1/3$, $(\sigma_{e0}^2, \sigma_{e1}^2, \sigma_{e2}^2) = (1, 1, 1)$ and $MR = \{0, 0.4\}$ for quantitative trait. The upper side and the right side of each subplot are respectively the distribution of the true value of γ and that of the point estimate of γ. (**a**) $\hat{\gamma}_{BNM}$ with $MR = 0$; (**b**) $\hat{\gamma}_{BNM}$ with $MR = 0.4$; (**c**) $\hat{\gamma}_{BUM}$ with $MR = 0$; (**d**) $\hat{\gamma}_{BUM}$ with $MR = 0.4$; (**e**) $\hat{\gamma}_{BNP}$ with $MR = 0$; (**f**) $\hat{\gamma}_{BNP}$ with $MR = 0.4$; (**g**) $\hat{\gamma}_{BUP}$ with $MR = 0$; (**h**) $\hat{\gamma}_{BUP}$ with $MR = 0.4$; (**i**) $\hat{\gamma}_{BN}$ with $MR = 0$; (**j**) $\hat{\gamma}_{BN}$ with $MR = 0.4$; (**k**) $\hat{\gamma}_{BU}$ with $MR = 0$; (**l**) $\hat{\gamma}_{BU}$ with $MR = 0.4$.

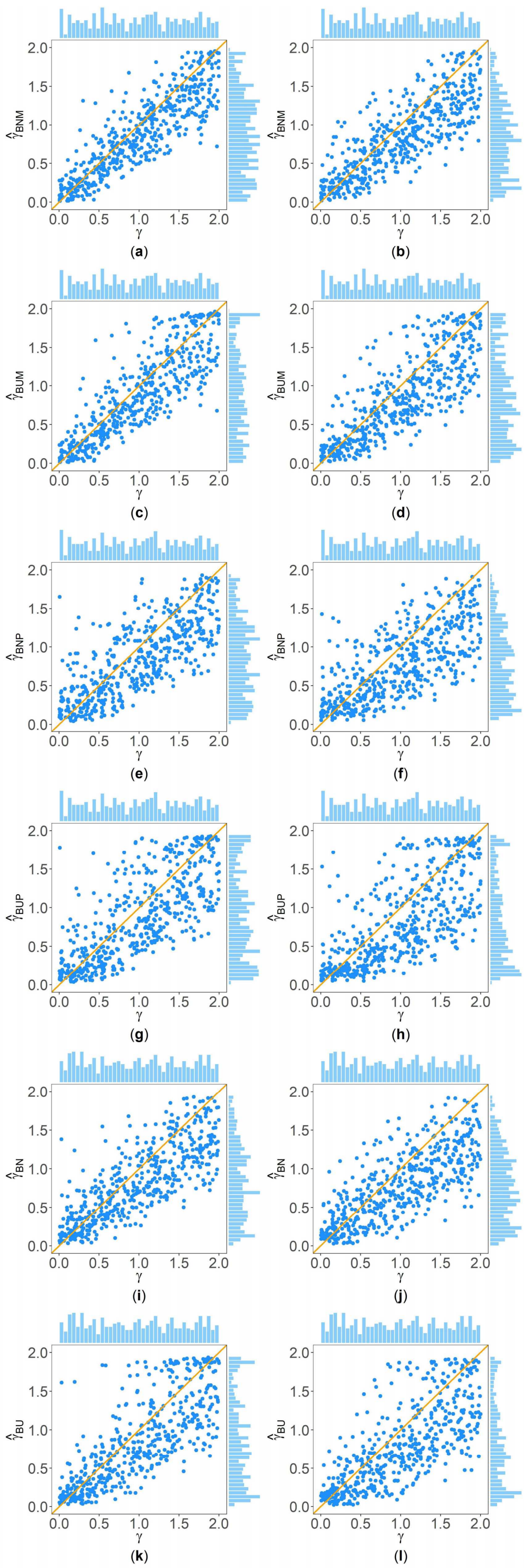

Figure S7. Scatter plots of six point estimates of γ against true values of γ with $N_p = 600$, $n_{If} = 2600$, $p_f = p_m = 0.1$, $\sigma_g^2 = 1$, $(\sigma_{e0}^2, \sigma_{e1}^2, \sigma_{e2}^2) = (1, 1, 1)$ and $MR = \{0, 0.4\}$ for quantitative trait. The upper side and the right side of each subplot are respectively the distribution of the true value of γ and that of the point estimate of γ. (**a**) $\hat{\gamma}_{BNM}$ with $MR = 0$; (**b**) $\hat{\gamma}_{BNM}$ with $MR = 0.4$; (**c**) $\hat{\gamma}_{BUM}$ with $MR = 0$; (**d**) $\hat{\gamma}_{BUM}$ with $MR = 0.4$; (**e**) $\hat{\gamma}_{BNP}$ with $MR = 0$; (**f**) $\hat{\gamma}_{BNP}$ with $MR = 0.4$; (**g**) $\hat{\gamma}_{BUP}$ with $MR = 0$; (**h**) $\hat{\gamma}_{BUP}$ with $MR = 0.4$; (**i**) $\hat{\gamma}_{BN}$ with $MR = 0$; (**j**) $\hat{\gamma}_{BN}$ with $MR = 0.4$; (**k**) $\hat{\gamma}_{BU}$ with $MR = 0$; (**l**) $\hat{\gamma}_{BU}$ with $MR = 0.4$.

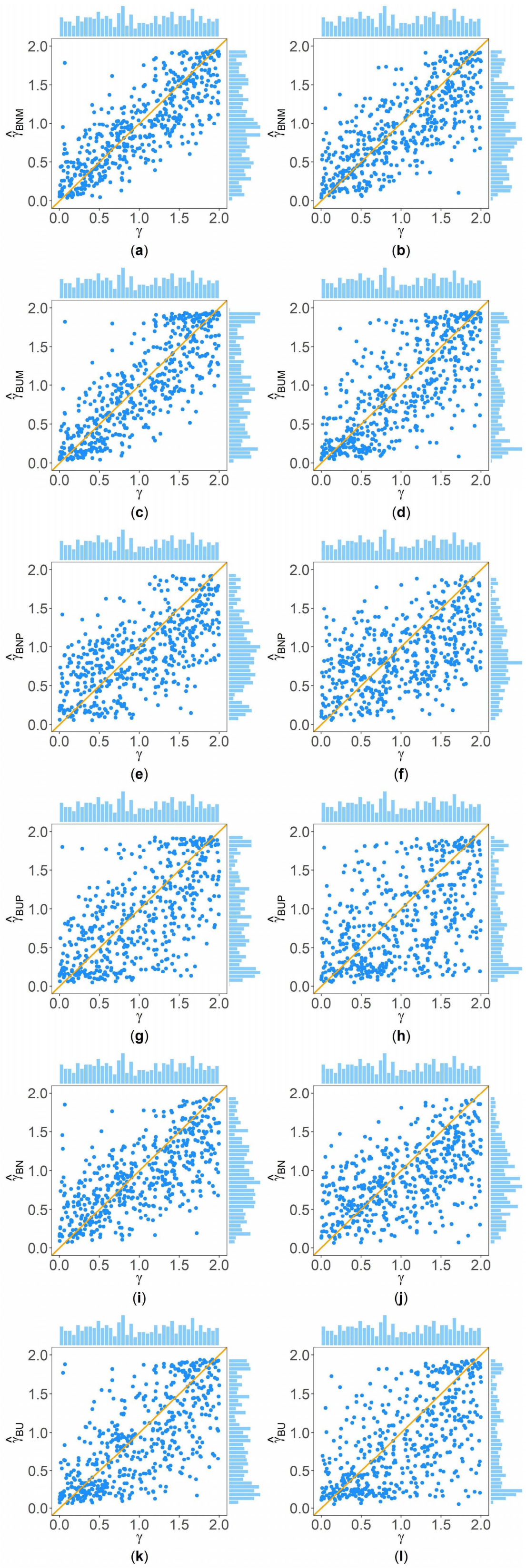

Figure S8. Scatter plots of six point estimates of γ against true values of γ with $N_p = 150$, $n_{If} = 650$, $p_f = p_m = 0.3$, $\sigma_g^2 = 1$, $(\sigma_{e0}^2, \sigma_{e1}^2, \sigma_{e2}^2) = (1, 1, 1)$ and $MR = \{0, 0.4\}$ for qualitative trait. The upper side and the right side of each subplot are respectively the distribution of the true value of γ and that of the point estimate of γ. (**a**) $\hat{\gamma}_{BNM}$ with $MR = 0$; (**b**) $\hat{\gamma}_{BNM}$ with $MR = 0.4$; (**c**) $\hat{\gamma}_{BUM}$ with $MR = 0$; (**d**) $\hat{\gamma}_{BUM}$ with $MR = 0.4$; (**e**) $\hat{\gamma}_{BNP}$ with $MR = 0$; (**f**) $\hat{\gamma}_{BNP}$ with $MR = 0.4$; (**g**) $\hat{\gamma}_{BUP}$ with $MR = 0$; (**h**) $\hat{\gamma}_{BUP}$ with $MR = 0.4$; (**i**) $\hat{\gamma}_{BN}$ with $MR = 0$; (**j**) $\hat{\gamma}_{BN}$ with $MR = 0.4$; (**k**) $\hat{\gamma}_{BU}$ with $MR = 0$; (**l**) $\hat{\gamma}_{BU}$ with $MR = 0.4$.

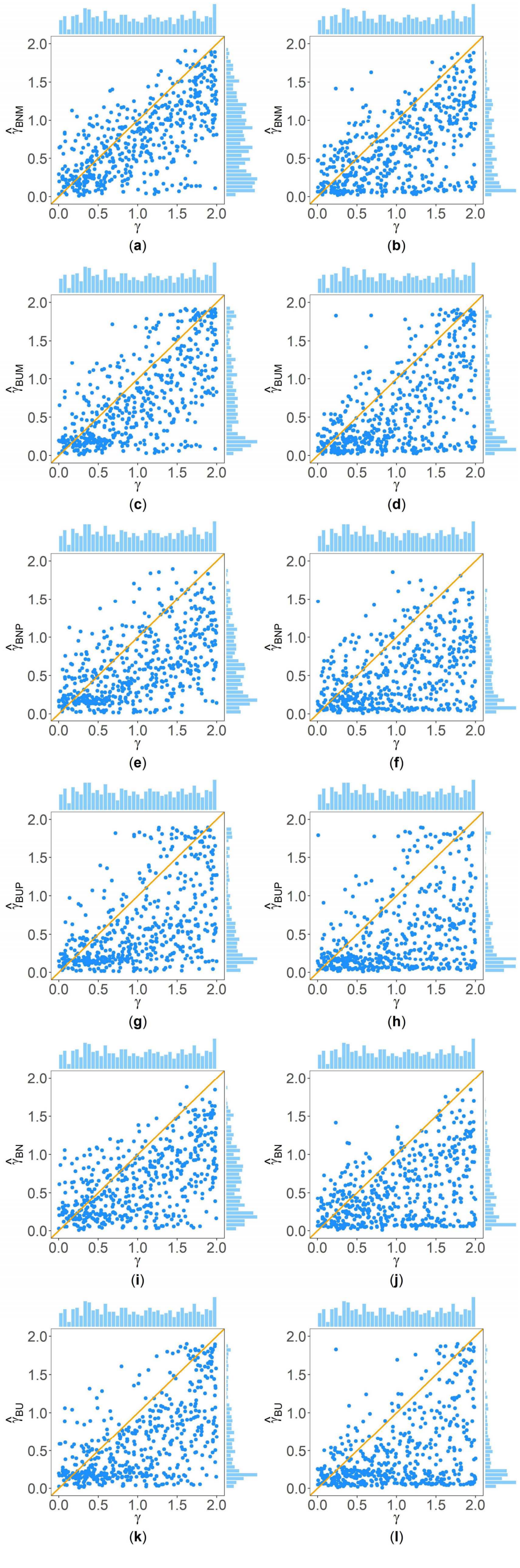

Figure S9. Scatter plots of six point estimates of γ against true values of γ with $N_p = 150$, $n_{If} = 650$, $p_f = p_m = 0.1$, $\sigma_g^2 = 1/3$, $(\sigma_{e0}^2, \sigma_{e1}^2, \sigma_{e2}^2) = (1, 1, 1)$ and $MR = \{0, 0.4\}$ for qualitative trait. The upper side and the right side of each subplot are respectively the distribution of the true value of γ and that of the point estimate of γ. (**a**) $\hat{\gamma}_{BNM}$ with $MR = 0$; (**b**) $\hat{\gamma}_{BNM}$ with $MR = 0.4$; (**c**) $\hat{\gamma}_{BUM}$ with $MR = 0$; (**d**) $\hat{\gamma}_{BUM}$ with $MR = 0.4$; (**e**) $\hat{\gamma}_{BNP}$ with $MR = 0$; (**f**) $\hat{\gamma}_{BNP}$ with $MR = 0.4$; (**g**) $\hat{\gamma}_{BUP}$ with $MR = 0$; (**h**) $\hat{\gamma}_{BUP}$ with $MR = 0.4$; (**i**) $\hat{\gamma}_{BN}$ with $MR = 0$; (**j**) $\hat{\gamma}_{BN}$ with $MR = 0.4$; (**k**) $\hat{\gamma}_{BU}$ with $MR = 0$; (**l**) $\hat{\gamma}_{BU}$ with $MR = 0.4$.

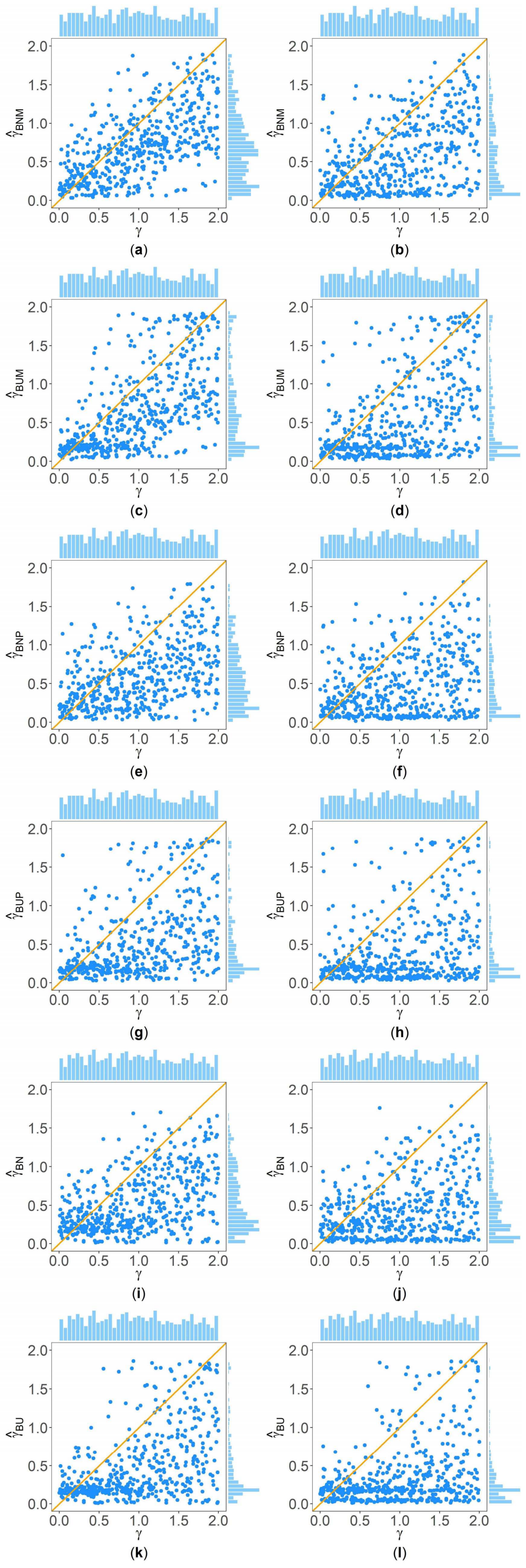

Figure S10. Scatter plots of six point estimates of γ against true values of γ with $N_p = 150$, $n_{If} = 650$, $p_f = p_m = 0.1$, $\sigma_g^2 = 1$, $(\sigma_{e0}^2, \sigma_{e1}^2, \sigma_{e2}^2) = (1, 1, 1)$ and $MR = \{0, 0.4\}$ for qualitative trait. The upper side and the right side of each subplot are respectively the distribution of the true value of γ and that of the point estimate of γ. (**a**) $\hat{\gamma}_{BNM}$ with $MR = 0$; (**b**) $\hat{\gamma}_{BNM}$ with $MR = 0.4$; (**c**) $\hat{\gamma}_{BUM}$ with $MR = 0$; (**d**) $\hat{\gamma}_{BUM}$ with $MR = 0.4$; (**e**) $\hat{\gamma}_{BNP}$ with $MR = 0$; (**f**) $\hat{\gamma}_{BNP}$ with $MR = 0.4$; (**g**) $\hat{\gamma}_{BUP}$ with $MR = 0$; (**h**) $\hat{\gamma}_{BUP}$ with $MR = 0.4$; (**i**) $\hat{\gamma}_{BN}$ with $MR = 0$; (**j**) $\hat{\gamma}_{BN}$ with $MR = 0.4$; (**k**) $\hat{\gamma}_{BU}$ with $MR = 0$; (**l**) $\hat{\gamma}_{BU}$ with $MR = 0.4$.

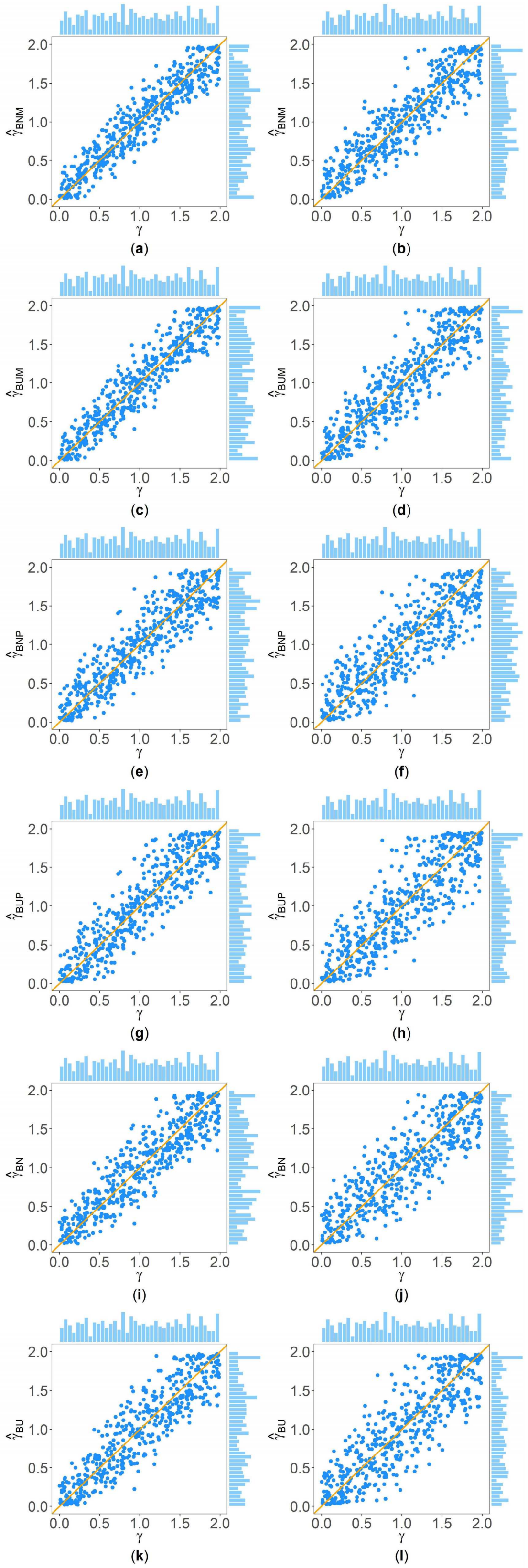

Figure S11. Scatter plots of six point estimates of γ against true values of γ with $N_p = 600$, $n_{If} = 2600$, $p_f = p_m = 0.3$, $\sigma_g^2 = 1/3$, $(\sigma_{e0}^2, \sigma_{e1}^2, \sigma_{e2}^2) = (1, 1, 1)$ and $MR = \{0, 0.4\}$ for qualitative trait. The upper side and the right side of each subplot are respectively the distribution of the true value of γ and that of the point estimate of γ. (**a**) $\hat{\gamma}_{BNM}$ with $MR = 0$; (**b**) $\hat{\gamma}_{BNM}$ with $MR = 0.4$; (**c**) $\hat{\gamma}_{BUM}$ with $MR = 0$; (**d**) $\hat{\gamma}_{BUM}$ with $MR = 0.4$; (**e**) $\hat{\gamma}_{BNP}$ with $MR = 0$; (**f**) $\hat{\gamma}_{BNP}$ with $MR = 0.4$; (**g**) $\hat{\gamma}_{BUP}$ with $MR = 0$; (**h**) $\hat{\gamma}_{BUP}$ with $MR = 0.4$; (**i**) $\hat{\gamma}_{BN}$ with $MR = 0$; (**j**) $\hat{\gamma}_{BN}$ with $MR = 0.4$; (**k**) $\hat{\gamma}_{BU}$ with $MR = 0$; (**l**) $\hat{\gamma}_{BU}$ with $MR = 0.4$.

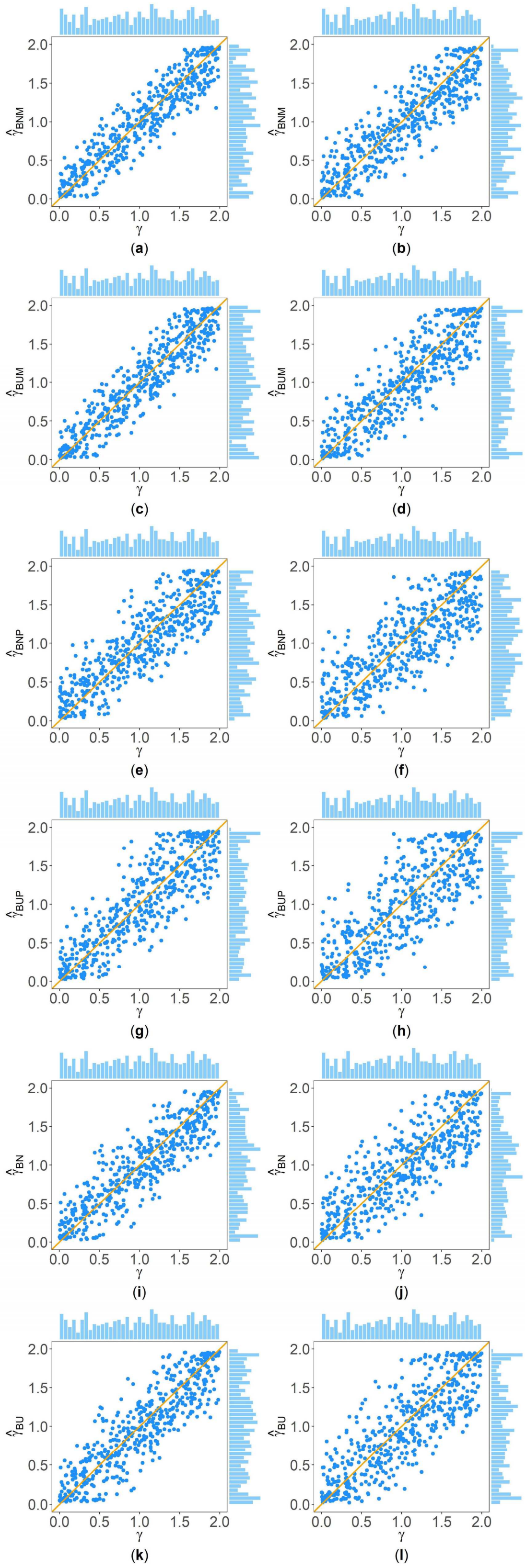

Figure S12. Scatter plots of six point estimates of γ against true values of γ with $N_p = 600$, $n_{If} = 2600$, $p_f = p_m = 0.3$, $\sigma_g^2 = 1$, $(\sigma_{e0}^2, \sigma_{e1}^2, \sigma_{e2}^2) = (1, 1, 1)$ and $MR = \{0, 0.4\}$ for qualitative trait. The upper side and the right side of each subplot are respectively the distribution of the true value of γ and that of the point estimate of γ. (**a**) $\hat{\gamma}_{BNM}$ with $MR = 0$; (**b**) $\hat{\gamma}_{BNM}$ with $MR = 0.4$; (**c**) $\hat{\gamma}_{BUM}$ with $MR = 0$; (**d**) $\hat{\gamma}_{BUM}$ with $MR = 0.4$; (**e**) $\hat{\gamma}_{BNP}$ with $MR = 0$; (**f**) $\hat{\gamma}_{BNP}$ with $MR = 0.4$; (**g**) $\hat{\gamma}_{BUP}$ with $MR = 0$; (**h**) $\hat{\gamma}_{BUP}$ with $MR = 0.4$; (**i**) $\hat{\gamma}_{BN}$ with $MR = 0$; (**j**) $\hat{\gamma}_{BN}$ with $MR = 0.4$; (**k**) $\hat{\gamma}_{BU}$ with $MR = 0$; (**l**) $\hat{\gamma}_{BU}$ with $MR = 0.4$.

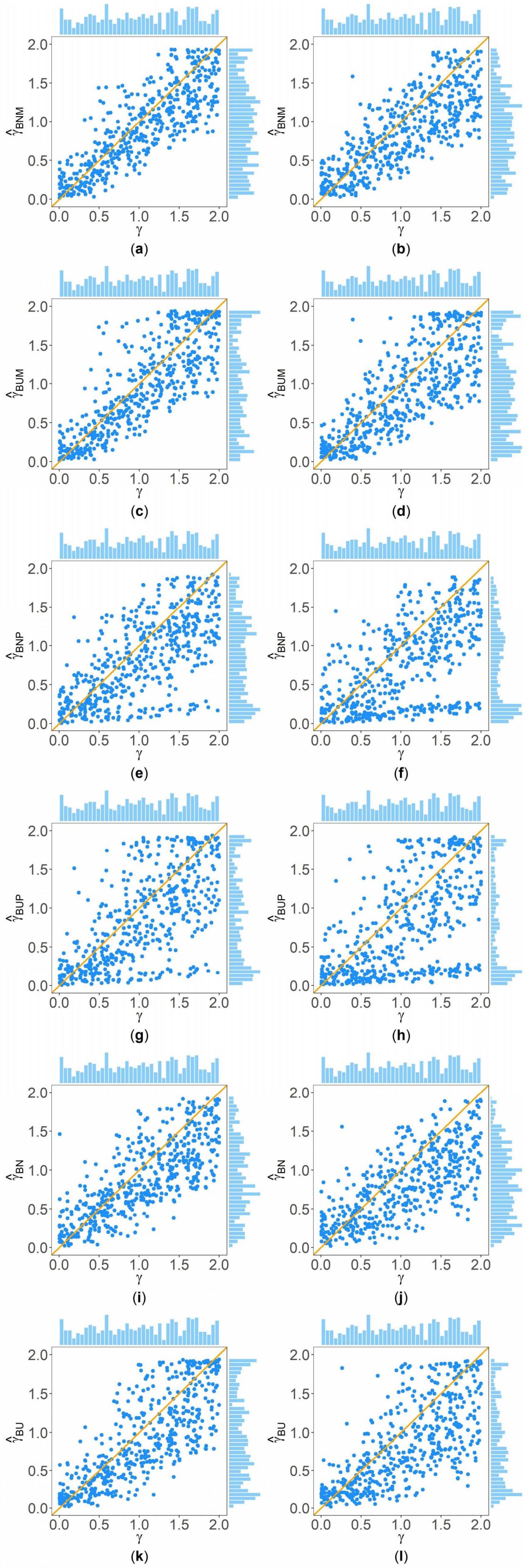

Figure S13. Scatter plots of six point estimates of γ against true values of γ with $N_p = 600$, $n_{If} = 2600$, $p_f = p_m = 0.1$, $\sigma_g^2 = 1/3$, $(\sigma_{e0}^2, \sigma_{e1}^2, \sigma_{e2}^2) = (1, 1, 1)$ and $MR = \{0, 0.4\}$ for qualitative trait. The upper side and the right side of each subplot are respectively the distribution of the true value of γ and that of the point estimate of γ. (**a**) $\hat{\gamma}_{BNM}$ with $MR = 0$; (**b**) $\hat{\gamma}_{BNM}$ with $MR = 0.4$; (**c**) $\hat{\gamma}_{BUM}$ with $MR = 0$; (**d**) $\hat{\gamma}_{BUM}$ with $MR = 0.4$; (**e**) $\hat{\gamma}_{BNP}$ with $MR = 0$; (**f**) $\hat{\gamma}_{BNP}$ with $MR = 0.4$; (**g**) $\hat{\gamma}_{BUP}$ with $MR = 0$; (**h**) $\hat{\gamma}_{BUP}$ with $MR = 0.4$; (**i**) $\hat{\gamma}_{BN}$ with $MR = 0$; (**j**) $\hat{\gamma}_{BN}$ with $MR = 0.4$; (**k**) $\hat{\gamma}_{BU}$ with $MR = 0$; (**l**) $\hat{\gamma}_{BU}$ with $MR = 0.4$.

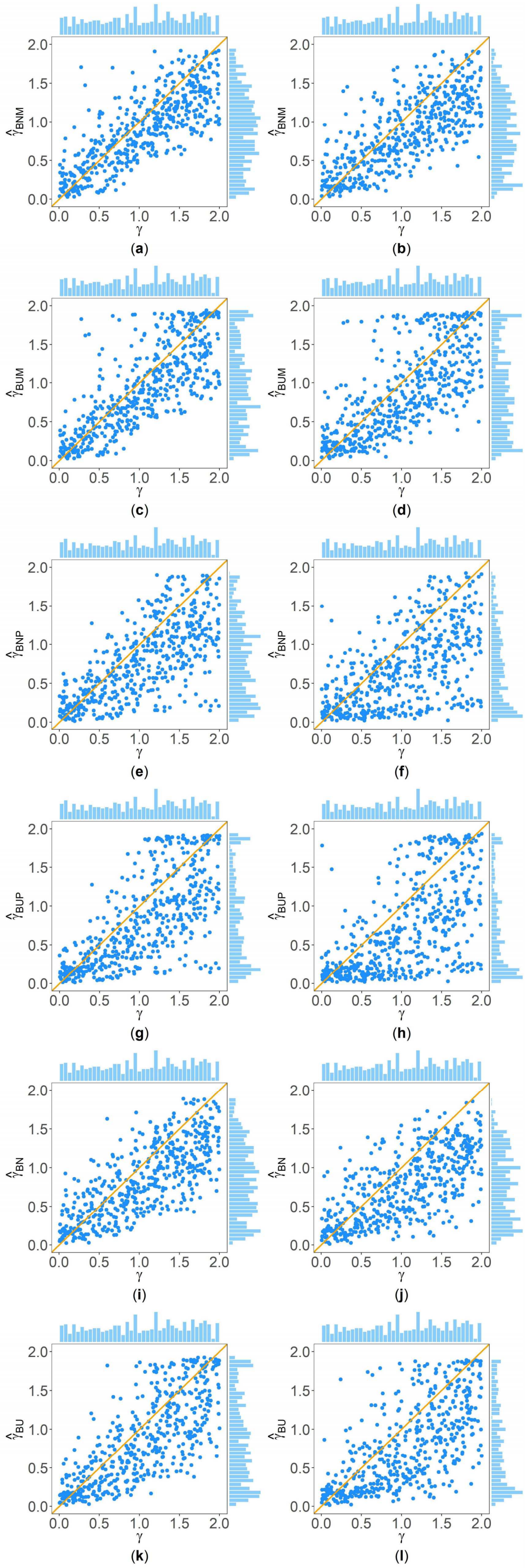

Figure S14. Scatter plots of six point estimates of γ against true values of γ with $N_p = 600$, $n_{If} = 2600$, $p_f = p_m = 0.1$, $\sigma_g^2 = 1$, $(\sigma_{e0}^2, \sigma_{e1}^2, \sigma_{e2}^2) = (1,1,1)$ and $MR = \{0, 0.4\}$ for qualitative trait. The upper side and the right side of each subplot are respectively the distribution of the true value of γ and that of the point estimate of γ. (**a**) $\hat{\gamma}_{BNM}$ with $MR = 0$; (**b**) $\hat{\gamma}_{BNM}$ with $MR = 0.4$; (**c**) $\hat{\gamma}_{BUM}$ with $MR = 0$; (**d**) $\hat{\gamma}_{BUM}$ with $MR = 0.4$; (**e**) $\hat{\gamma}_{BNP}$ with $MR = 0$; (**f**) $\hat{\gamma}_{BNP}$ with $MR = 0.4$; (**g**) $\hat{\gamma}_{BUP}$ with $MR = 0$; (**h**) $\hat{\gamma}_{BUP}$ with $MR = 0.4$; (**i**) $\hat{\gamma}_{BN}$ with $MR = 0$; (**j**) $\hat{\gamma}_{BN}$ with $MR = 0.4$; (**k**) $\hat{\gamma}_{BU}$ with $MR = 0$; (**l**) $\hat{\gamma}_{BU}$ with $MR = 0.4$.

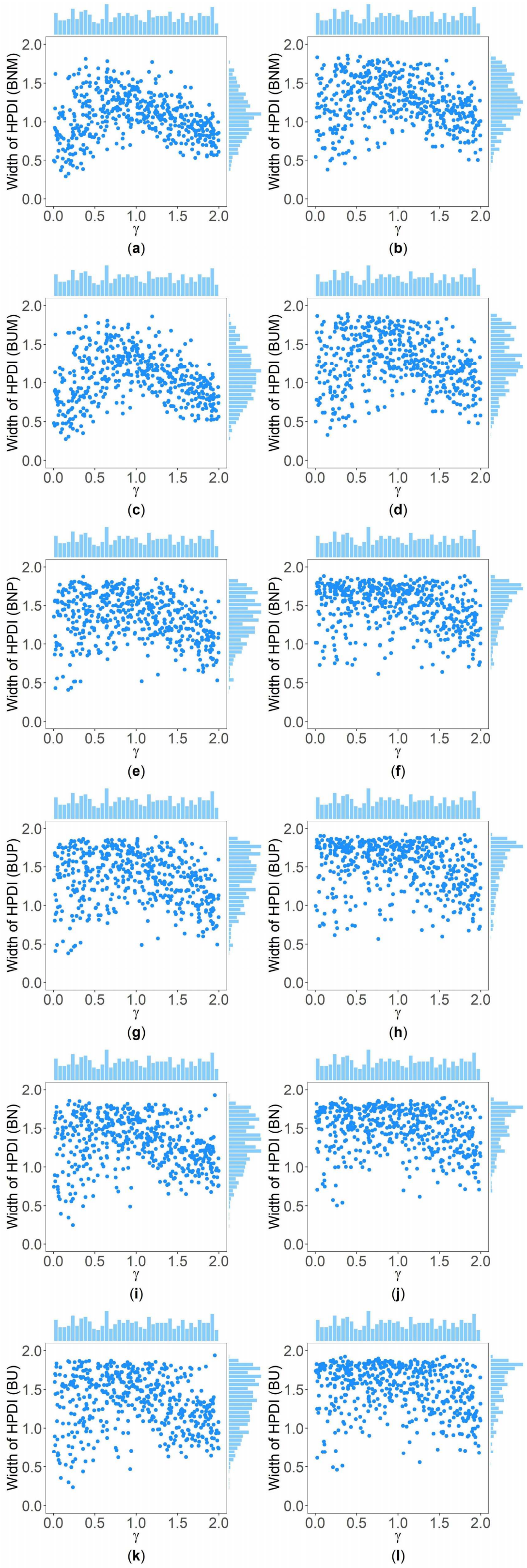

Figure S15. Scatter plots of widths of HPDIs based on six methods against true values of γ with $N_p = 150$, $n_{If} = 650$, $p_f = p_m = 0.3$, $\sigma_g^2 = 1$, $(\sigma_{e0}^2, \sigma_{e1}^2, \sigma_{e2}^2) = (1, 1, 1)$ and $MR = \{0, 0.4\}$ for quantitative trait. The upper side and the right side of each subplot are respectively the distribution of the true value of γ and that of the width of the HPDI of γ. (**a**) BNM with $MR = 0$; (**b**) BNM with $MR = 0.4$; (**c**) BUM with $MR = 0$; (**d**) BUM with $MR = 0.4$; (**e**) BNP with $MR = 0$; (**f**) BNP with $MR = 0.4$; (**g**) BUP with $MR = 0$; (**h**) BUP with $MR = 0.4$; (**i**) BN with $MR = 0$; (**j**) BN with $MR = 0.4$; (**k**) BU with $MR = 0$; (**l**) BU with $MR = 0.4$.

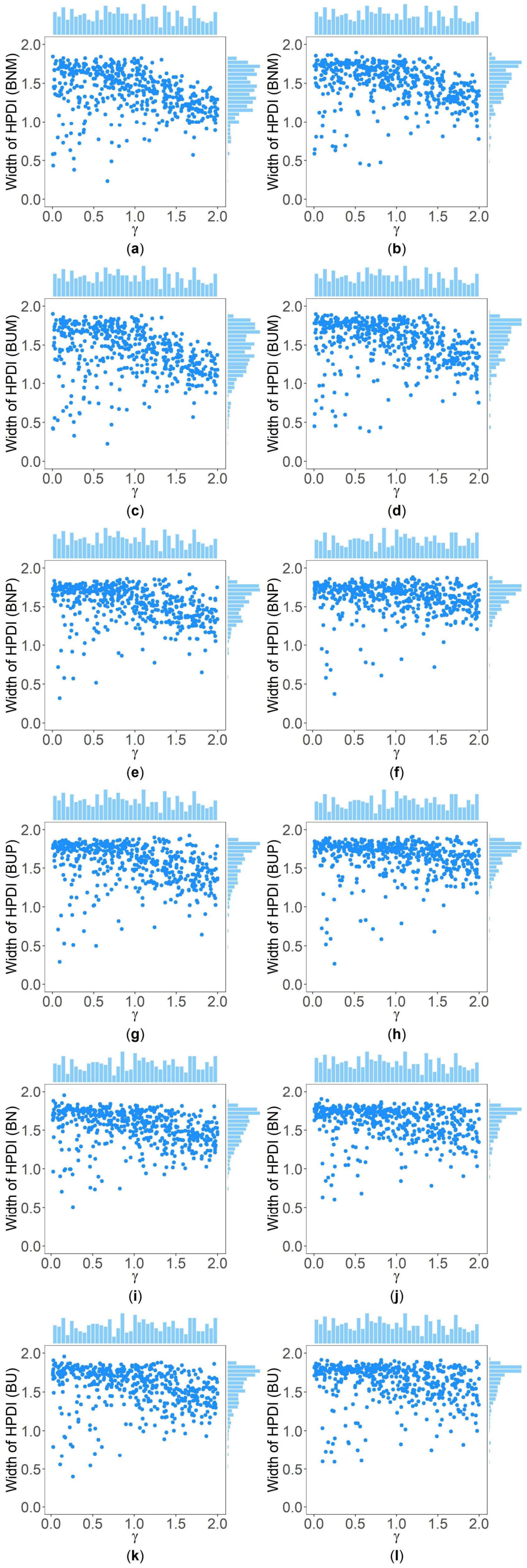

Figure S16. Scatter plots of widths of HPDIs based on six methods against true values of γ with $N_p = 150$, $n_{If} = 650$, $p_f = p_m = 0.1$, $\sigma_g^2 = 1/3$, $(\sigma_{e0}^2, \sigma_{e1}^2, \sigma_{e2}^2) = (1, 1, 1)$ and $MR = \{0, 0.4\}$ for quantitative trait. The upper side and the right side of each subplot are respectively the distribution of the true value of γ and that of the width of the HPDI of γ. (**a**) BNM with $MR = 0$; (**b**) BNM with $MR = 0.4$; (**c**) BUM with $MR = 0$; (**d**) BUM with $MR = 0.4$; (**e**) BNP with $MR = 0$; (**f**) BNP with $MR = 0.4$; (**g**) BUP with $MR = 0$; (**h**) BUP with $MR = 0.4$; (**i**) BN with $MR = 0$; (**j**) BN with $MR = 0.4$; (**k**) BU with $MR = 0$; (**l**) BU with $MR = 0.4$.

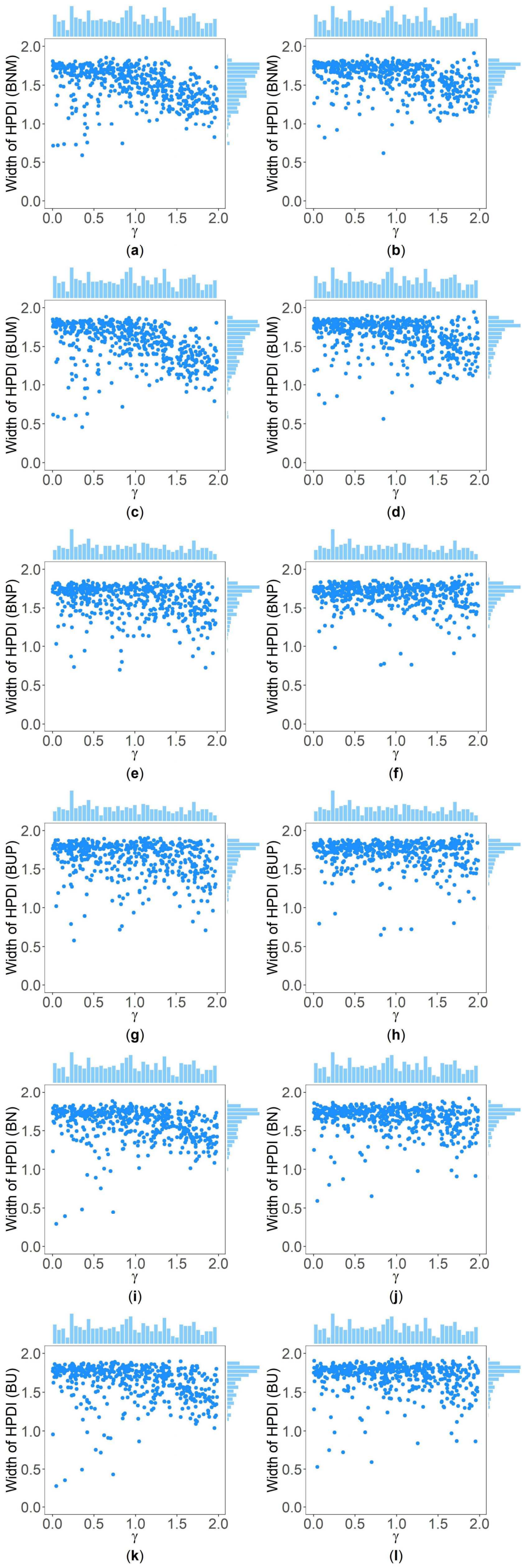

Figure S17. Scatter plots of widths of HPDIs based on six methods against true values of γ with $N_p = 150$, $n_{If} = 650$, $p_f = p_m = 0.1$, $\sigma_g^2 = 1$, $(\sigma_{e0}^2, \sigma_{e1}^2, \sigma_{e2}^2) = (1, 1, 1)$ and $MR = \{0, 0.4\}$ for quantitative trait. The upper side and the right side of each subplot are respectively the distribution of the true value of γ and that of the width of the HPDI of γ. (**a**) BNM with $MR = 0$; (**b**) BNM with $MR = 0.4$; (**c**) BUM with $MR = 0$; (**d**) BUM with $MR = 0.4$; (**e**) BNP with $MR = 0$; (**f**) BNP with $MR = 0.4$; (**g**) BUP with $MR = 0$; (**h**) BUP with $MR = 0.4$; (**i**) BN with $MR = 0$; (**j**) BN with $MR = 0.4$; (**k**) BU with $MR = 0$; (**l**) BU with $MR = 0.4$.

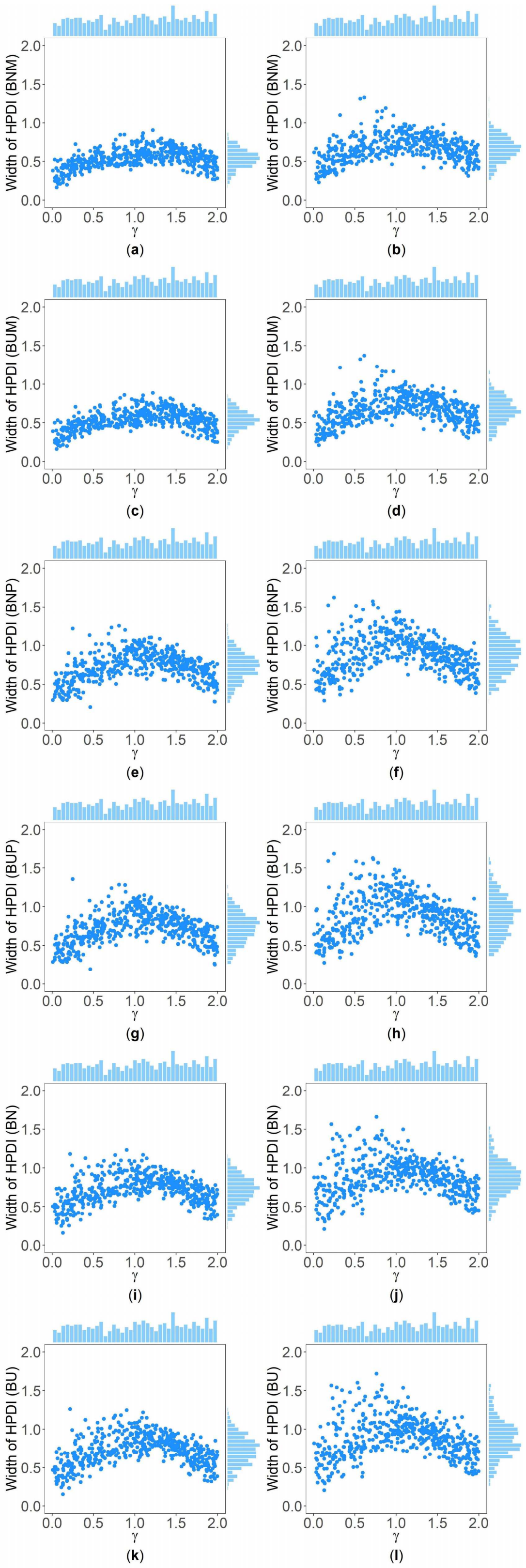

Figure S18. Scatter plots of widths of HPDIs based on six methods against true values of γ with $N_p = 600$, $n_{If} = 2600$, $p_f = p_m = 0.3$, $\sigma_g^2 = 1/3$, $(\sigma_{e0}^2, \sigma_{e1}^2, \sigma_{e2}^2) = (1, 1, 1)$ and $MR = \{0, 0.4\}$ for quantitative trait. The upper side and the right side of each subplot are respectively the distribution of the true value of γ and that of the width of the HPDI of γ. (**a**) BNM with $MR = 0$; (**b**) BNM with $MR = 0.4$; (**c**) BUM with $MR = 0$; (**d**) BUM with $MR = 0.4$; (**e**) BNP with $MR = 0$; (**f**) BNP with $MR = 0.4$; (**g**) BUP with $MR = 0$; (**h**) BUP with $MR = 0.4$; (**i**) BN with $MR = 0$; (**j**) BN with $MR = 0.4$; (**k**) BU with $MR = 0$; (**l**) BU with $MR = 0.4$.

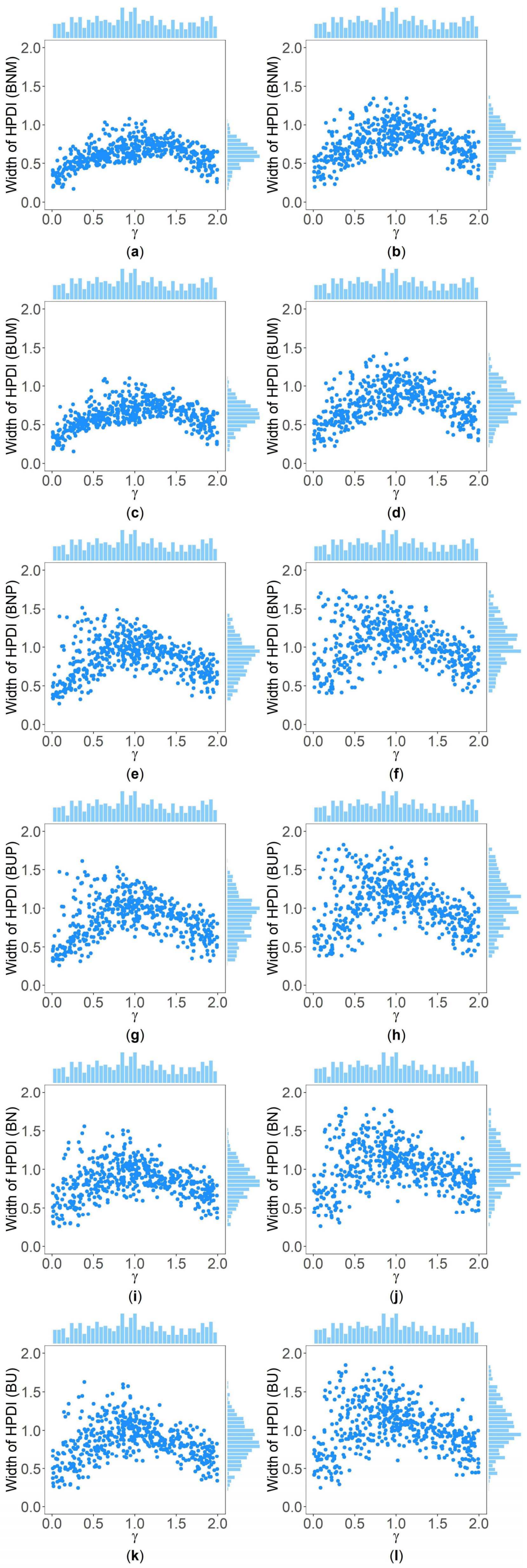

Figure S19. Scatter plots of widths of HPDIs based on six methods against true values of γ with $N_p = 600$, $n_{If} = 2600$, $p_f = p_m = 0.3$, $\sigma_g^2 = 1$, $(\sigma_{e0}^2, \sigma_{e1}^2, \sigma_{e2}^2) = (1, 1, 1)$ and $MR = \{0, 0.4\}$ for quantitative trait. The upper side and the right side of each subplot are respectively the distribution of the true value of γ and that of the width of the HPDI of γ. (**a**) BNM with $MR = 0$; (**b**) BNM with $MR = 0.4$; (**c**) BUM with $MR = 0$; (**d**) BUM with $MR = 0.4$; (**e**) BNP with $MR = 0$; (**f**) BNP with $MR = 0.4$; (**g**) BUP with $MR = 0$; (**h**) BUP with $MR = 0.4$; (**i**) BN with $MR = 0$; (**j**) BN with $MR = 0.4$; (**k**) BU with $MR = 0$; (**l**) BU with $MR = 0.4$.

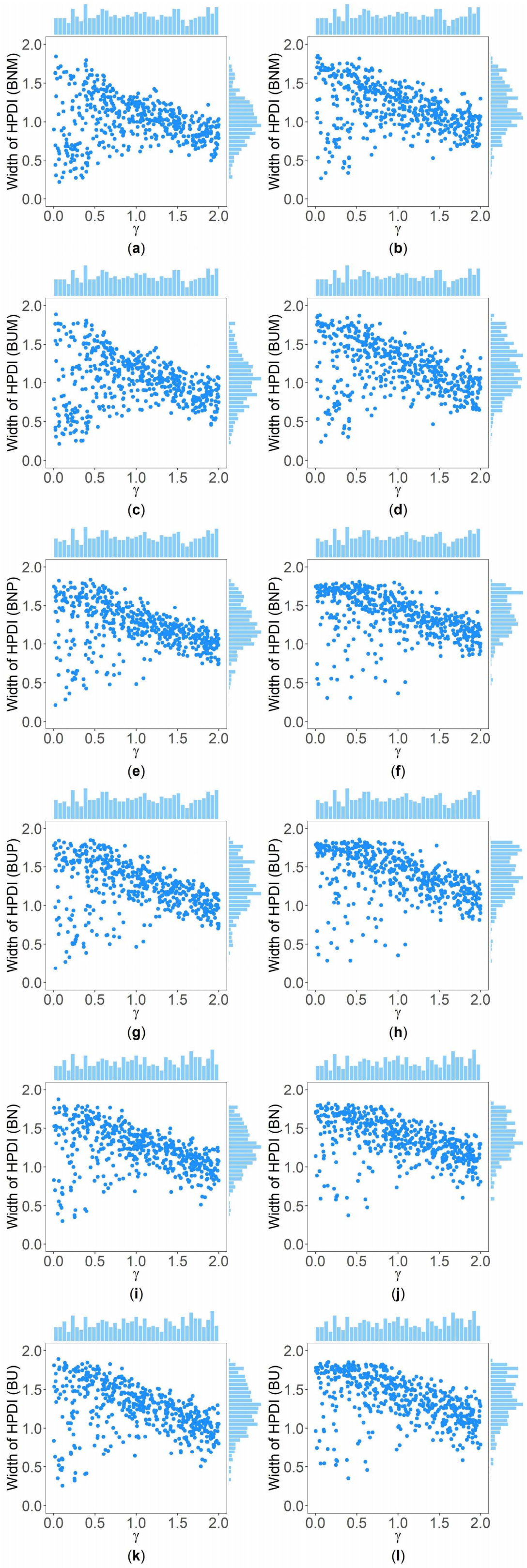

Figure S20. Scatter plots of widths of HPDIs based on six methods against true values of γ with $N_p = 600$, $n_{If} = 2600$, $p_f = p_m = 0.1$, $\sigma_g^2 = 1/3$, $(\sigma_{e0}^2, \sigma_{e1}^2, \sigma_{e2}^2) = (1, 1, 1)$ and $MR = \{0, 0.4\}$ for quantitative trait. The upper side and the right side of each subplot are respectively the distribution of the true value of γ and that of the width of the HPDI of γ. (**a**) BNM with $MR = 0$; (**b**) BNM with $MR = 0.4$; (**c**) BUM with $MR = 0$; (**d**) BUM with $MR = 0.4$; (**e**) BNP with $MR = 0$; (**f**) BNP with $MR = 0.4$; (**g**) BUP with $MR = 0$; (**h**) BUP with $MR = 0.4$; (**i**) BN with $MR = 0$; (**j**) BN with $MR = 0.4$; (**k**) BU with $MR = 0$; (**l**) BU with $MR = 0.4$.

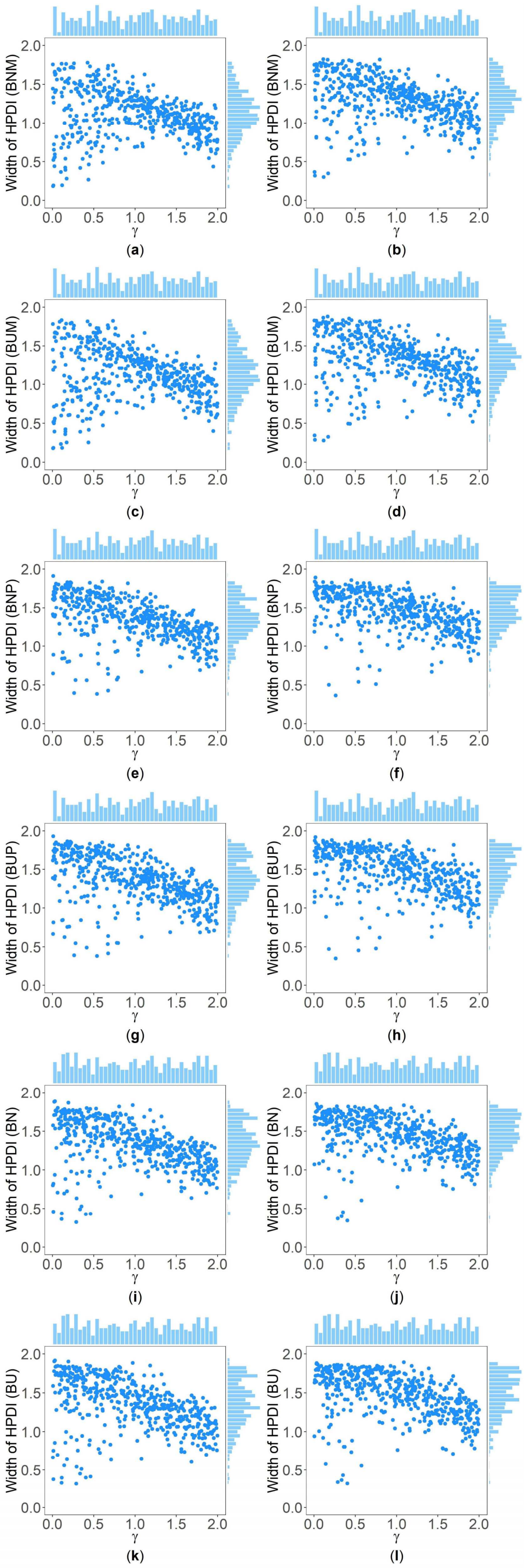

Figure S21. Scatter plots of widths of HPDIs based on six methods against true values of γ with $N_p = 600$, $n_{If} = 2600$, $p_f = p_m = 0.1$, $\sigma_g^2 = 1$, $(\sigma_{e0}^2, \sigma_{e1}^2, \sigma_{e2}^2) = (1, 1, 1)$ and $MR = \{0, 0.4\}$ for quantitative trait. The upper side and the right side of each subplot are respectively the distribution of the true value of γ and that of the width of the HPDI of γ. (**a**) BNM with $MR = 0$; (**b**) BNM with $MR = 0.4$; (**c**) BUM with $MR = 0$; (**d**) BUM with $MR = 0.4$; (**e**) BNP with $MR = 0$; (**f**) BNP with $MR = 0.4$; (**g**) BUP with $MR = 0$; (**h**) BUP with $MR = 0.4$; (**i**) BN with $MR = 0$; (**j**) BN with $MR = 0.4$; (**k**) BU with $MR = 0$; (**l**) BU with $MR = 0.4$.

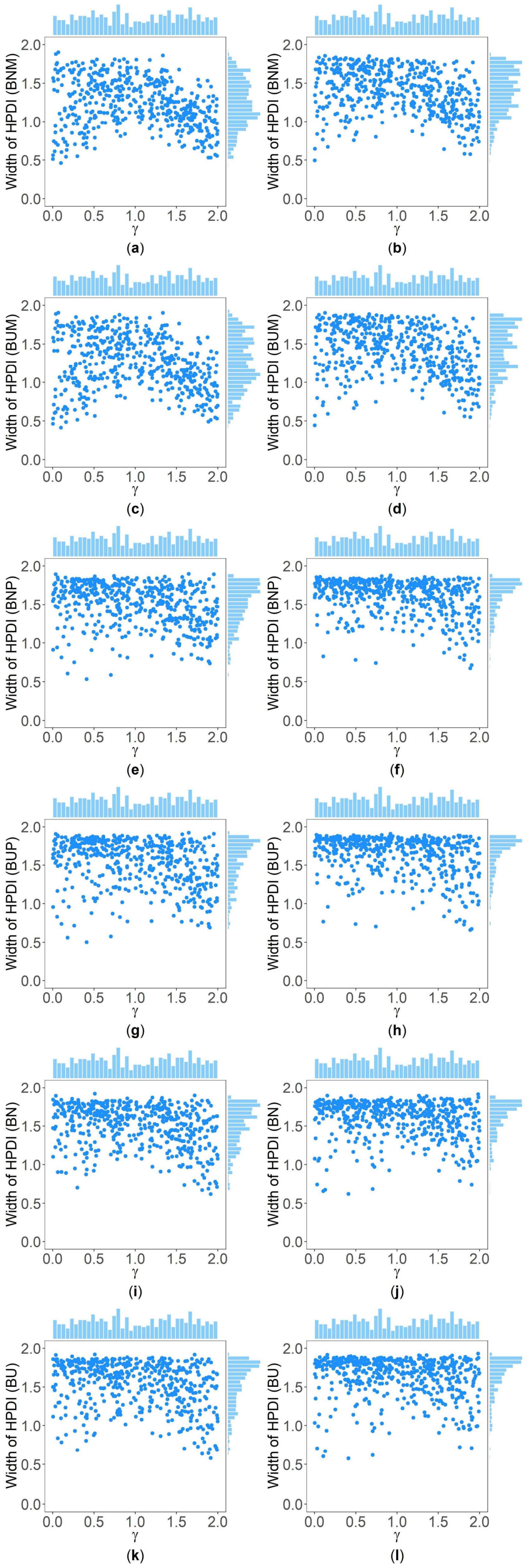

Figure S22. Scatter plots of widths of HPDIs based on six methods against true values of γ with $N_p = 150$, $n_{If} = 650$, $p_f = p_m = 0.3$, $\sigma_g^2 = 1$, $(\sigma_{e0}^2, \sigma_{e1}^2, \sigma_{e2}^2) = (1, 1, 1)$ and $MR = \{0, 0.4\}$ for qualitative trait. The upper side and the right side of each subplot are respectively the distribution of the true value of γ and that of the width of the HPDI of γ. (**a**) BNM with $MR = 0$; (**b**) BNM with $MR = 0.4$; (**c**) BUM with $MR = 0$; (**d**) BUM with $MR = 0.4$; (**e**) BNP with $MR = 0$; (**f**) BNP with $MR = 0.4$; (**g**) BUP with $MR = 0$; (**h**) BUP with $MR = 0.4$; (**i**) BN with $MR = 0$; (**j**) BN with $MR = 0.4$; (**k**) BU with $MR = 0$; (**l**) BU with $MR = 0.4$.

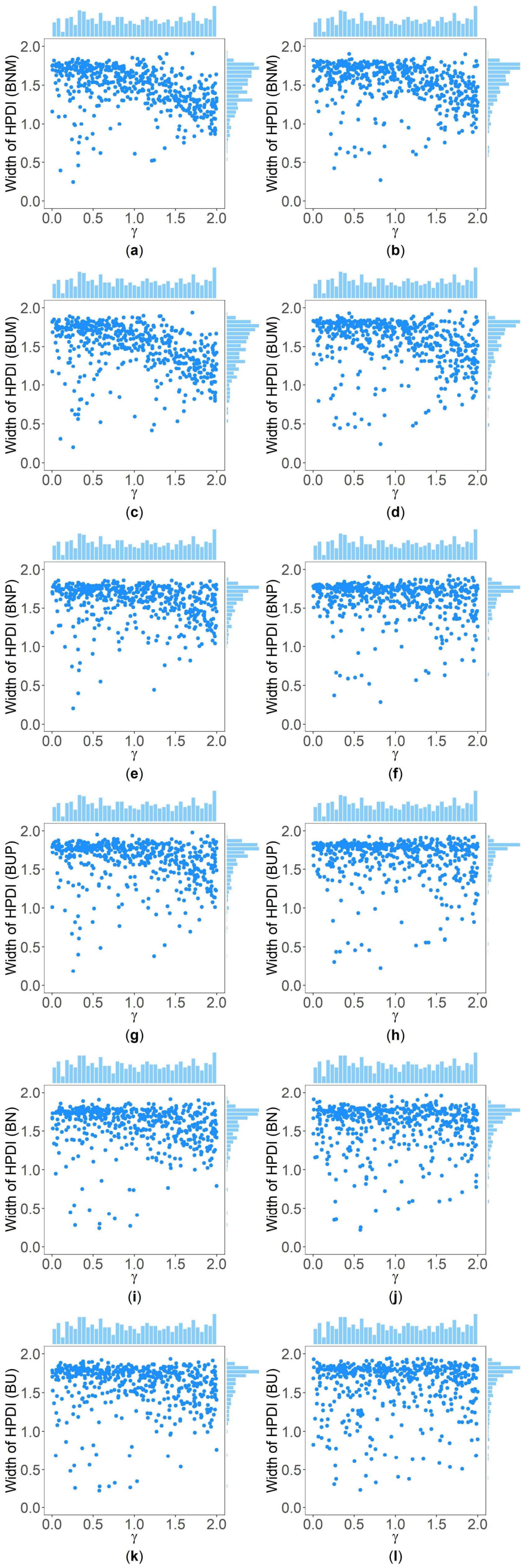

Figure S23. Scatter plots of widths of HPDIs based on six methods against true values of γ with $N_p = 150$, $n_{If} = 650$, $p_f = p_m = 0.1$, $\sigma_g^2 = 1/3$, $(\sigma_{e0}^2, \sigma_{e1}^2, \sigma_{e2}^2) = (1, 1, 1)$ and $MR = \{0, 0.4\}$ for qualitative trait. The upper side and the right side of each subplot are respectively the distribution of the true value of γ and that of the width of the HPDI of γ. (**a**) BNM with $MR = 0$; (**b**) BNM with $MR = 0.4$; (**c**) BUM with $MR = 0$; (**d**) BUM with $MR = 0.4$; (**e**) BNP with $MR = 0$; (**f**) BNP with $MR = 0.4$; (**g**) BUP with $MR = 0$; (**h**) BUP with $MR = 0.4$; (**i**) BN with $MR = 0$; (**j**) BN with $MR = 0.4$; (**k**) BU with $MR = 0$; (**l**) BU with $MR = 0.4$.

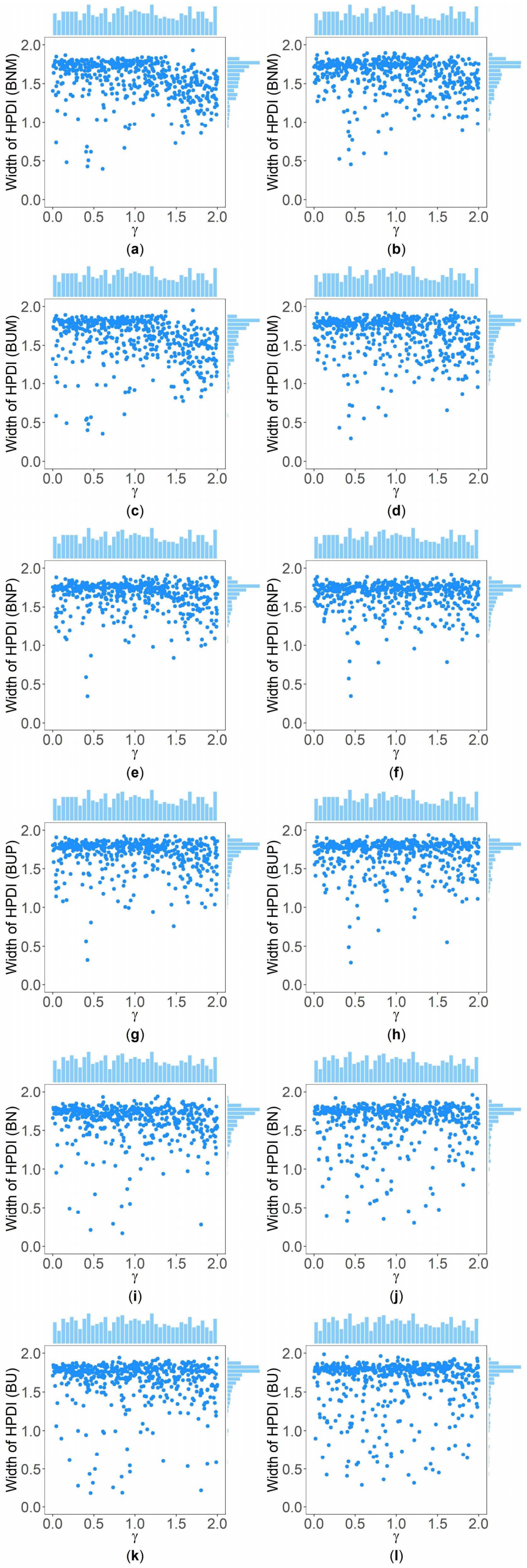

Figure S24. Scatter plots of widths of HPDIs based on six methods against true values of γ with $N_p = 150$, $n_{If} = 650$, $p_f = p_m = 0.1$, $\sigma_g^2 = 1$, $(\sigma_{e0}^2, \sigma_{e1}^2, \sigma_{e2}^2) = (1, 1, 1)$ and $MR = \{0, 0.4\}$ for qualitative trait. The upper side and the right side of each subplot are respectively the distribution of the true value of γ and that of the width of the HPDI of γ. (**a**) BNM with $MR = 0$; (**b**) BNM with $MR = 0.4$; (**c**) BUM with $MR = 0$; (**d**) BUM with $MR = 0.4$; (**e**) BNP with $MR = 0$; (**f**) BNP with $MR = 0.4$; (**g**) BUP with $MR = 0$; (**h**) BUP with $MR = 0.4$; (**i**) BN with $MR = 0$; (**j**) BN with $MR = 0.4$; (**k**) BU with $MR = 0$; (**l**) BU with $MR = 0.4$.

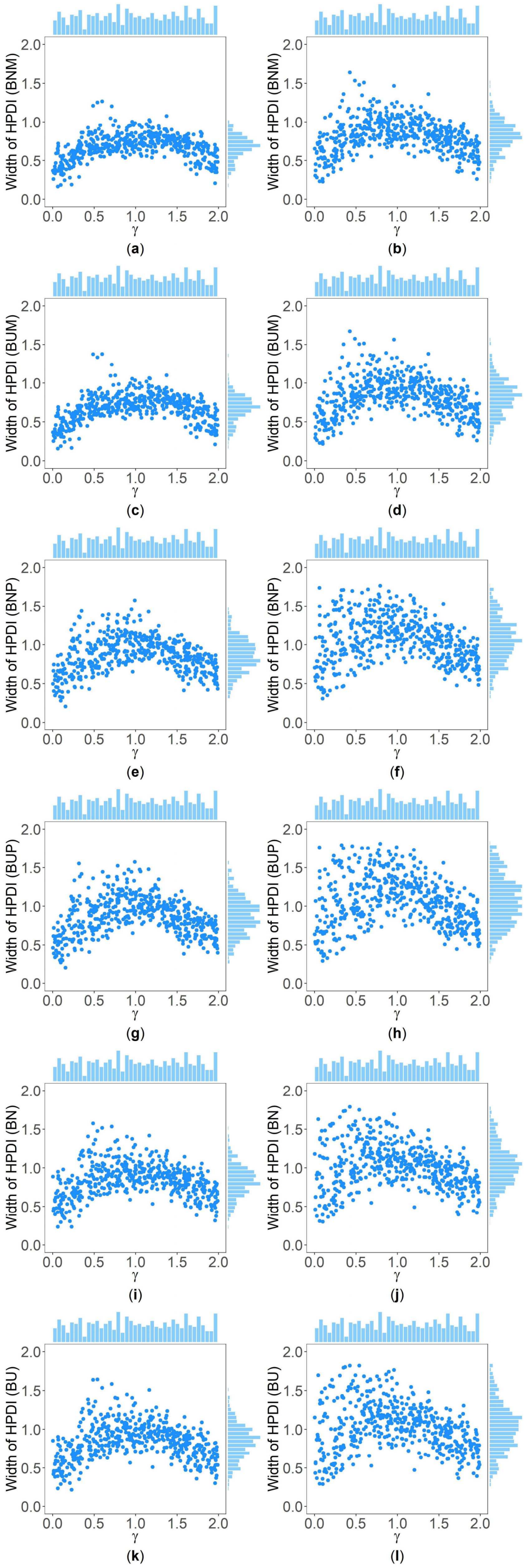

Figure S25. Scatter plots of widths of HPDIs based on six methods against true values of γ with $N_p = 600$, $n_{If} = 2600$, $p_f = p_m = 0.3$, $\sigma_g^2 = 1/3$, $(\sigma_{e0}^2, \sigma_{e1}^2, \sigma_{e2}^2) = (1, 1, 1)$ and $MR = \{0, 0.4\}$ for qualitative trait. The upper side and the right side of each subplot are respectively the distribution of the true value of γ and that of the width of the HPDI of γ. (**a**) BNM with $MR = 0$; (**b**) BNM with $MR = 0.4$; (**c**) BUM with $MR = 0$; (**d**) BUM with $MR = 0.4$; (**e**) BNP with $MR = 0$; (**f**) BNP with $MR = 0.4$; (**g**) BUP with $MR = 0$; (**h**) BUP with $MR = 0.4$; (**i**) BN with $MR = 0$; (**j**) BN with $MR = 0.4$; (**k**) BU with $MR = 0$; (**l**) BU with $MR = 0.4$.

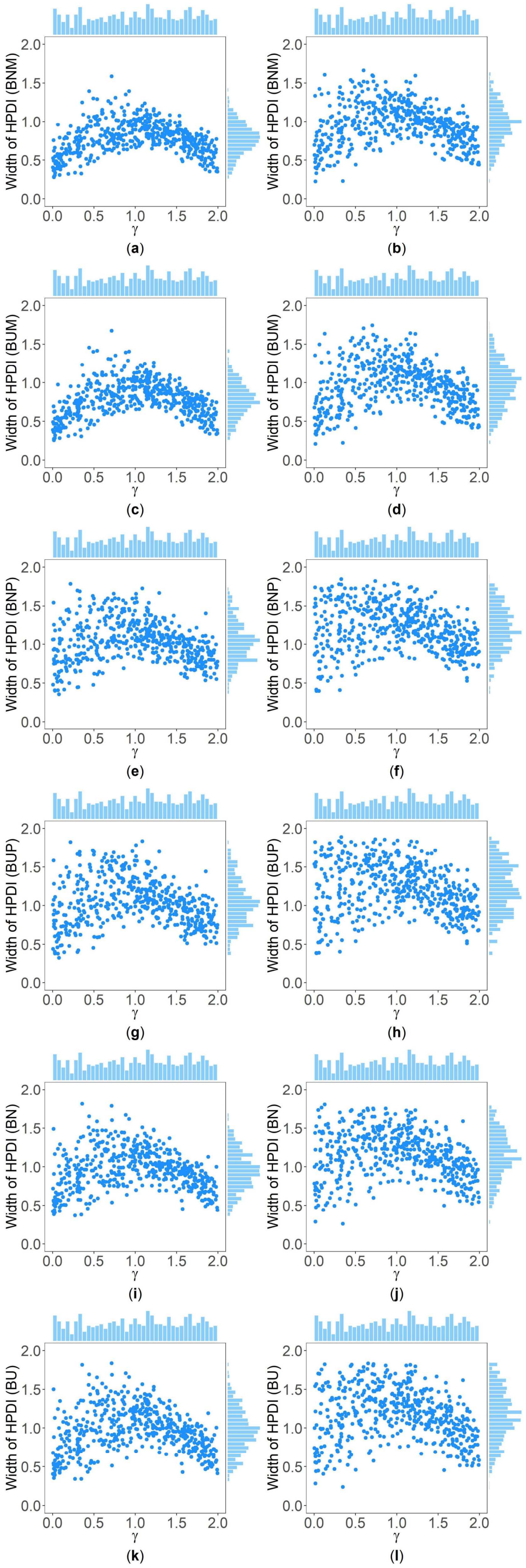

Figure S26. Scatter plots of widths of HPDIs based on six methods against true values of γ with $N_p = 600$, $n_{If} = 2600$, $p_f = p_m = 0.3$, $\sigma_g^2 = 1$, $(\sigma_{e0}^2, \sigma_{e1}^2, \sigma_{e2}^2) = (1,1,1)$ and $MR = \{0, 0.4\}$ for qualitative trait. The upper side and the right side of each subplot are respectively the distribution of the true value of γ and that of the width of the HPDI of γ. (**a**) BNM with $MR = 0$; (**b**) BNM with $MR = 0.4$; (**c**) BUM with $MR = 0$; (**d**) BUM with $MR = 0.4$; (**e**) BNP with $MR = 0$; (**f**) BNP with $MR = 0.4$; (**g**) BUP with $MR = 0$; (**h**) BUP with $MR = 0.4$; (**i**) BN with $MR = 0$; (**j**) BN with $MR = 0.4$; (**k**) BU with $MR = 0$; (**l**) BU with $MR = 0.4$.

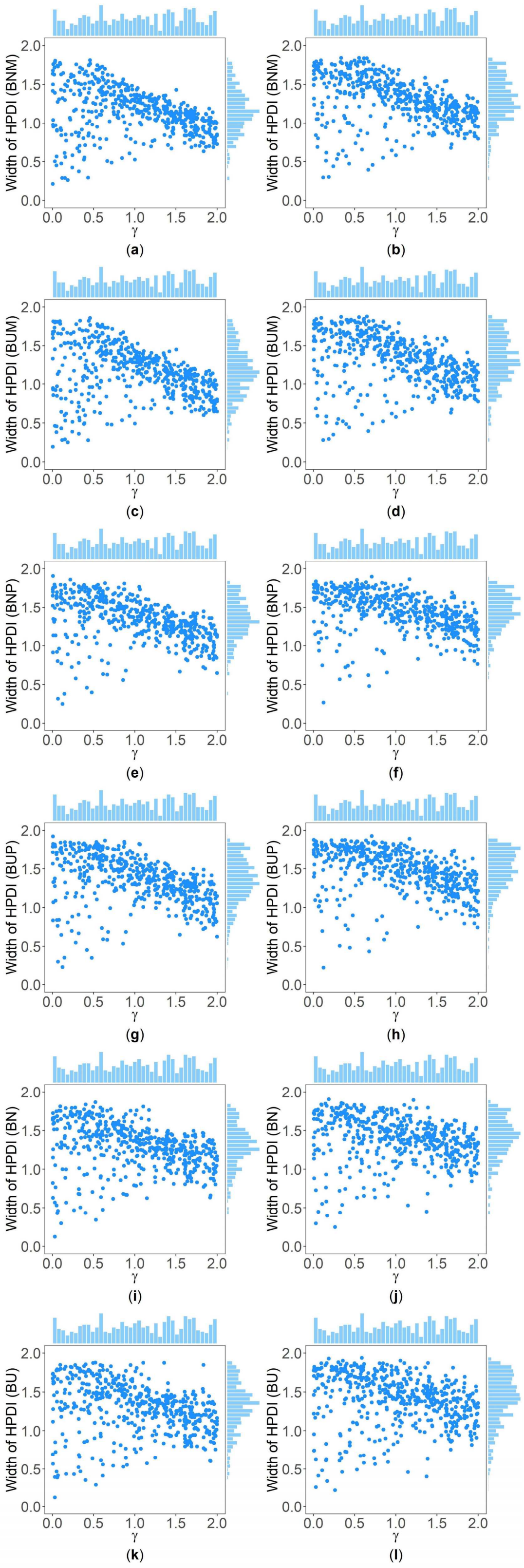

Figure S27. Scatter plots of widths of HPDIs based on six methods against true values of γ with $N_p = 600$, $n_{If} = 2600$, $p_f = p_m = 0.1$, $\sigma_g^2 = 1/3$, $(\sigma_{e0}^2, \sigma_{e1}^2, \sigma_{e2}^2) = (1, 1, 1)$ and $MR = \{0, 0.4\}$ for qualitative trait. The upper side and the right side of each subplot are respectively the distribution of the true value of γ and that of the width of the HPDI of γ. (**a**) BNM with $MR = 0$; (**b**) BNM with $MR = 0.4$; (**c**) BUM with $MR = 0$; (**d**) BUM with $MR = 0.4$; (**e**) BNP with $MR = 0$; (**f**) BNP with $MR = 0.4$; (**g**) BUP with $MR = 0$; (**h**) BUP with $MR = 0.4$; (**i**) BN with $MR = 0$; (**j**) BN with $MR = 0.4$; (**k**) BU with $MR = 0$; (**l**) BU with $MR = 0.4$.

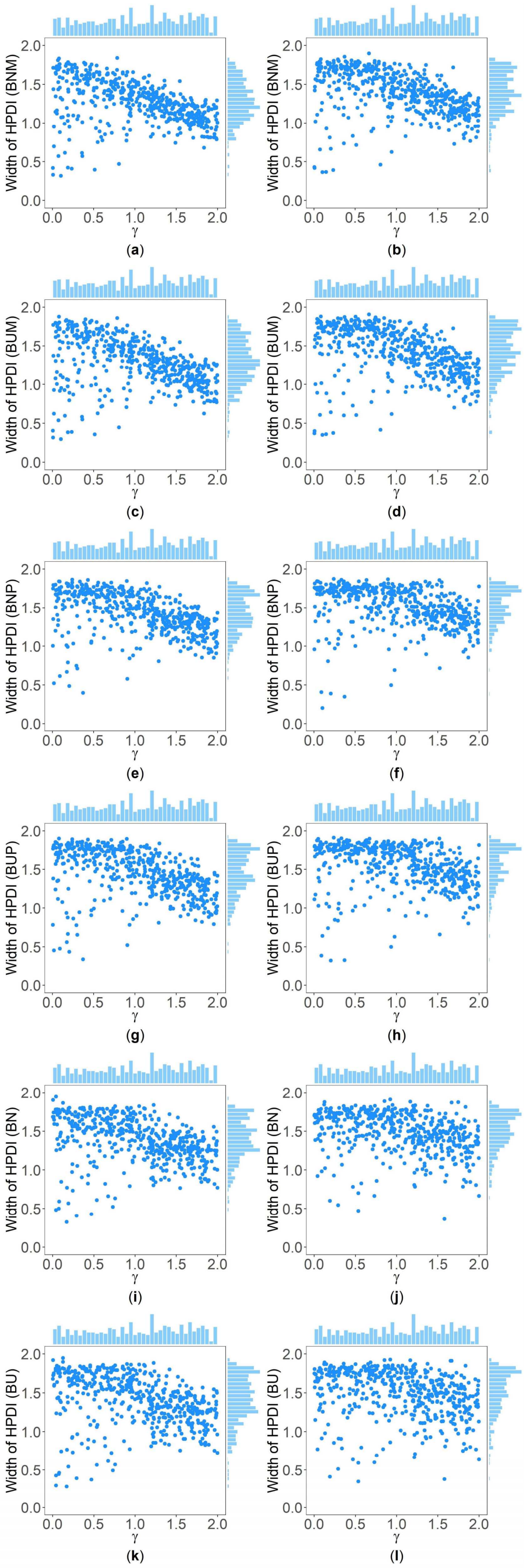

Figure S28. Scatter plots of widths of HPDIs based on six methods against true values of γ with $N_p = 600$, $n_{If} = 2600$, $p_f = p_m = 0.1$, $\sigma_g^2 = 1$, $(\sigma_{e0}^2, \sigma_{e1}^2, \sigma_{e2}^2) = (1, 1, 1)$ and $MR = \{0, 0.4\}$ for qualitative trait. The upper side and the right side of each subplot are respectively the distribution of the true value of γ and that of the width of the HPDI of γ. (**a**) BNM with $MR = 0$; (**b**) BNM with $MR = 0.4$; (**c**) BUM with $MR = 0$; (**d**) BUM with $MR = 0.4$; (**e**) BNP with $MR = 0$; (**f**) BNP with $MR = 0.4$; (**g**) BUP with $MR = 0$; (**h**) BUP with $MR = 0.4$; (**i**) BN with $MR = 0$; (**j**) BN with $MR = 0.4$; (**k**) BU with $MR = 0$; (**l**) BU with $MR = 0.4$.

Supplementary References

1. Runcie, D.E.; Crawford, L. Fast and flexible linear mixed models for genome-wide genetics. *PLoS Genet.* **2019**, *15*, e1007978.
2. Zhao, J.H.; Luan, J.A.; Congdon, P. Bayesian linear mixed models with polygenic effects. *J. Stat. Softw.* **2018**, *85*, 1-27.
